# Supplementary material for: Sleep Traits Causally Affect the Brain Cortical Structure: A Mendelian Randomization Study
Source: Biomedicines. 2023 Aug 18;11(8):2296. doi: 10.3390/biomedicines11082296 (PMC10452307; doi:10.3390/biomedicines11082296)
Supplement: Supplementary file 1 [file biomedicines-11-02296-s001.zip › supplementary figure(1).pdf]

Supplementary Information

Sleep traits causally affect the brain cortical structure: A Mendelian randomization study

Yanjing Chen[1], Shiyi Lyu[1], Wang Xiao[3], Sijie Yi[1], Ping Liu[1],Jun Liu[1,2]

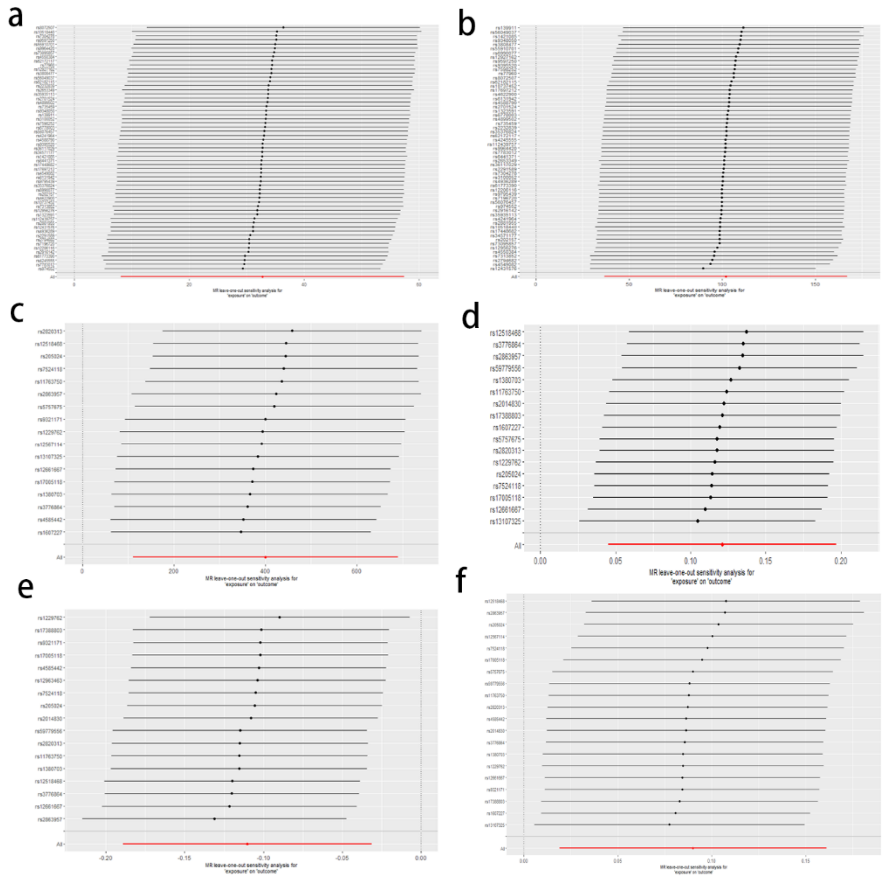

Figure S1. IVW estimates of stronger association results from morningness, long sleep, short sleep, ease of getting up and insomnia on cortical SA and TH with global weighted. (a) **Leave-one-out plots** from genetically predicted morningness on SA of the cuneus; (b) **Leave-one-out plots** from genetically predicted morningness on SA of the lateral occipital; (c) **Leave-one-out plots** from genetically predicted short sleep on SA of the lateral occipital; (d) **Leave-one-out plots** from genetically predicted short sleep on TH of the middle temporal; (e) **Leave-one-out plots** from genetically predicted short sleep on TH of paracenreal; (f) **Leave-one-out plots** from genetically predicted short sleep on TH of parahippocampal.

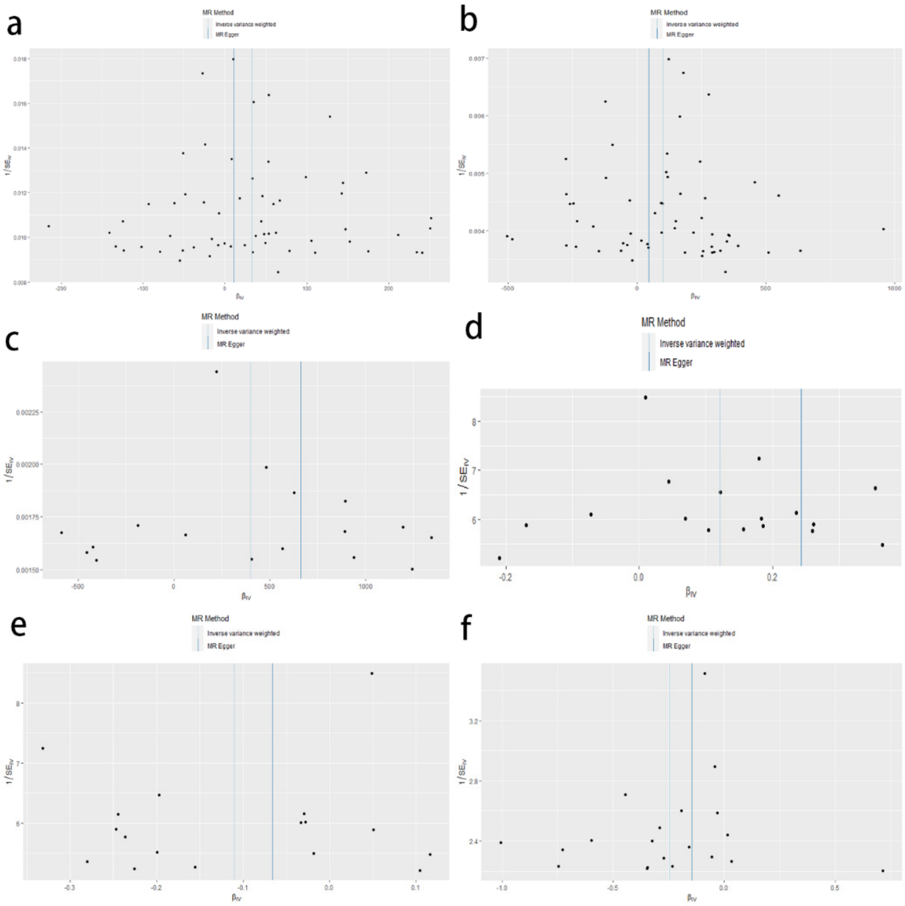

Figure S2. IVW estimates of stronger association results from morningness, long sleep, short sleep, ease of getting up and insomnia on cortical SA and TH with global weighted. (a) **Funnel plots** from genetically predicted morningness on SA of the cuneus; (b) **Funnel plots** from genetically predicted morningness on SA of the lateral occipital; (c) **Funnel plots** from genetically predicted short sleep on SA of the lateral occipital; (d) **Funnel plots** from genetically predicted short sleep on TH of the middle temporal; (e) **Funnel plots** from genetically predicted short sleep on TH of paracenreal; (f) **Funnel plots** from genetically predicted short sleep on TH of parahippocampal.

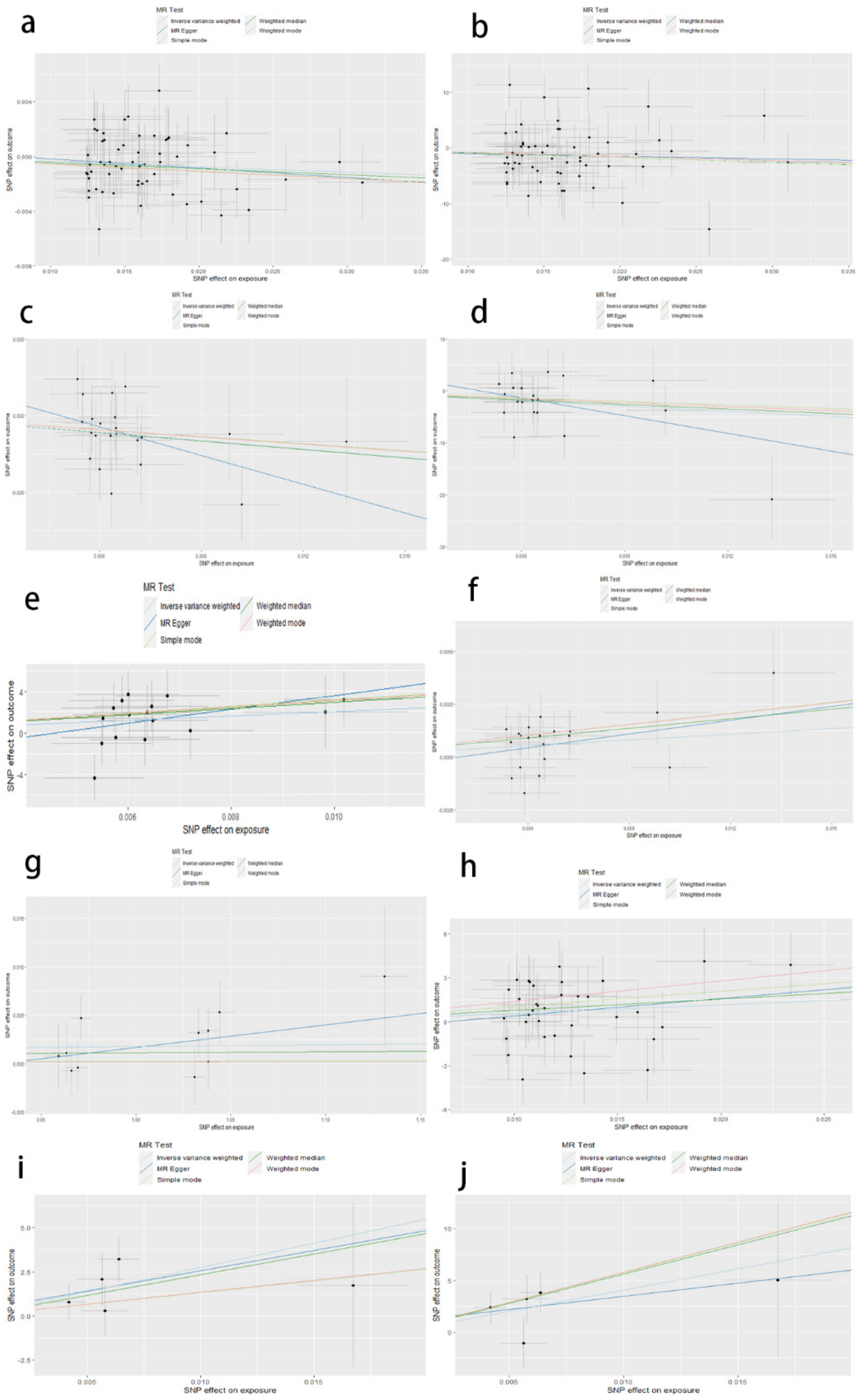

FigureS3. Scatter plots of stronger association and association estimates from genetically predicted (a) morningness on TH of the frontal pole with global weighted; (b) morningness on SA of the inferior parietal with global weighted; (c) short sleep on TH of the frontal pole with global weighted; (d) short sleep on SA of the inferior parietal with global weighted; (e) short sleep on SA of the middle temporal with global weighted; (f) short sleep on TH of the superior temporal with global weighted; (g) insomnia on TH of the parahippocampal with global weighted; (h) ease of getting up

on SA of the lateral orbitofrontal with global weighted; (i) long sleep on SA of isthmuscingulate with global weighted. (j) long sleep on SA of parsopercularis with global weighted.

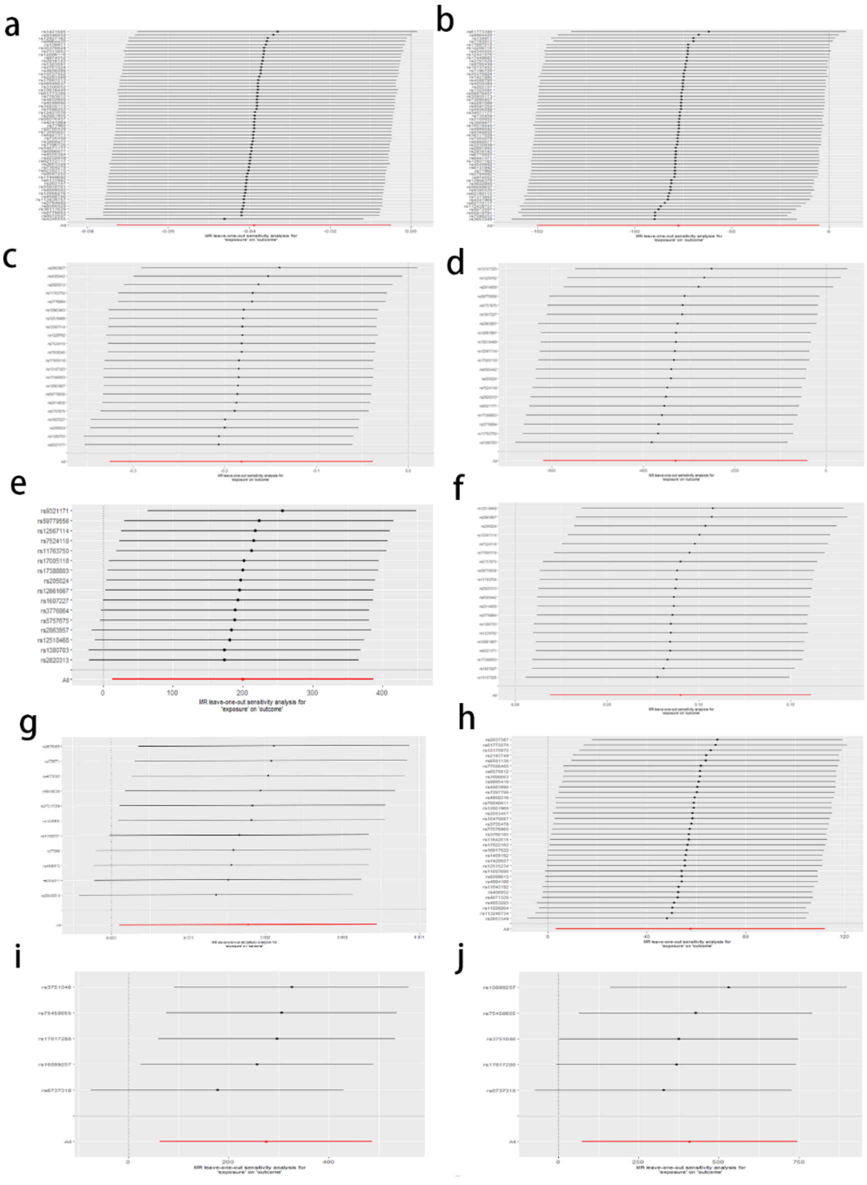

FigureS4. Leave-one-out plots of stronger association and association estimates from genetically predicted(a) morningness on TH of the frontal pole with global weighted; (b) morningness on SA of the inferior parietal with global weighted; (c) short sleep on TH of the frontal pole with global weighted; (d) short sleep on SA of the inferior parietal with global weighted; (e) short sleep on SA of the middle temporal with global weighted; (f) short sleep on TH of the superior temporal with global weighted; (g) insomnia on TH of the parahippocampal with global weighted; (h) ease of getting up on SA of the lateral orbitofrontal with global weighted; (i) long sleep on SA of isthmuscingulate with global weighted. (j) long sleep on SA of parsopercularis with global weighted.

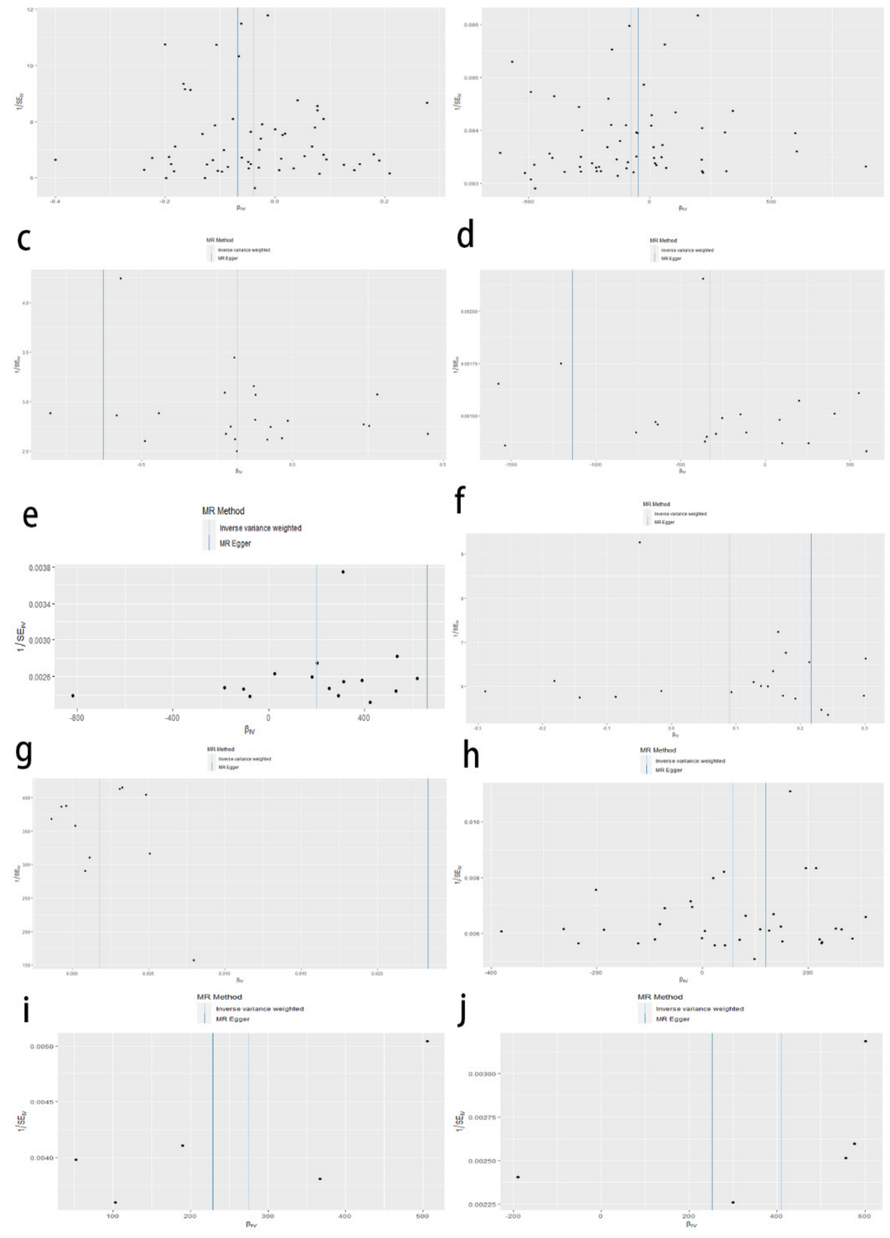

FigureS5. Funnel plots of stronger association and association estimates from genetically predicted (a) morningness on TH of the frontal pole with global weighted; (b) morningness on SA of the inferior parietal with global weighted; (c) short sleep on TH of the frontal pole with global weighted; (d) short sleep on SA of the inferior parietal with global weighted; (e) short sleep on SA of the middle temporal with global weighted; (f) short sleep on TH of the superior temporal with global weighted; (g) insomnia on TH of the parahippocampal with global weighted; (h) ease of getting up on SA of the lateral orbitofrontal with global weighted; (i) long sleep on SA of isthmuscingulate with global weighted. (j) long sleep on SA of parsopercularis with global weighted.
