# Supplementary material for: Sleep Traits Causally Affect the Brain Cortical Structure: A Mendelian Randomization Study
Source: Biomedicines. 2023 Aug 18;11(8):2296. doi: 10.3390/biomedicines11082296 (PMC10452307; doi:10.3390/biomedicines11082296)
Supplement: Supplementary file 1 [file biomedicines-11-02296-s001.zip › supplementary tables.pdf]

The details of genetic instruments predicting short sleep and cortical SA/TH														
**cortical SA/TH**	**Exposure**	**SNP**	**EA.exposure**	**OA.exposure**	**EA.outcome**	**OA.outcome**	**beta.exposure**	**se.exposure**	**pval.exposure**	**F**	**beta.outcome**	**se.outcome**	**pval.outcome**	**EAF**
**TH**	frontalpole	rs11763750	G	A	G	A	0.0072	0.0012	5.10E-09	34	-0.0032	0.0025	0.2002	0.81
**TH**	frontalpole	rs1229762	C	T	C	T	-0.0072	0.0010	1.00E-12	51	0.0014	0.0021	0.5191	0.34
**TH**	frontalpole	rs12518468	T	C	T	C	-0.0059	0.0010	8.50E-09	33	0.0013	0.0022	0.5426	0.67
**TH**	frontalpole	rs12567114	G	A	G	A	0.0063	0.0011	4.10E-09	34	-0.0013	0.0023	0.5660	0.72
**TH**	frontalpole	rs12661667	C	T	C	T	-0.0060	0.0011	2.80E-08	31	0.0005	0.0023	0.8202	0.74
**TH**	frontalpole	rs13107325	C	T	C	T	-0.0133	0.0018	2.50E-13	53	0.0017	0.0042	0.6882	0.93
**TH**	frontalpole	rs1380703	A	G	A	G	-0.0068	0.0010	1.60E-11	45	-0.0019	0.0022	0.3826	0.62
**TH**	frontalpole	rs1607227	G	T	G	T	0.0064	0.0011	1.50E-09	36	0.0015	0.0023	0.4995	0.70
**TH**	frontalpole	rs17005118	G	A	G	A	-0.0065	0.0011	2.50E-09	36	0.0008	0.0023	0.7364	0.74
**TH**	frontalpole	rs17388803	A	C	A	C	-0.0098	0.0016	6.50E-10	38	0.0012	0.0032	0.7142	0.89
**TH**	frontalpole	rs2014830	C	T	C	T	0.0058	0.0011	2.70E-08	30	-0.0002	0.0022	0.9373	0.70
**TH**	frontalpole	rs205024	C	T	C	T	0.0055	0.0010	2.70E-08	31	0.0014	0.0020	0.4901	0.62
**TH**	frontalpole	rs2820313	A	G	A	G	-0.0060	0.0010	2.30E-09	35	0.0035	0.0021	0.0974	0.66
**TH**	frontalpole	rs2863957	C	A	C	A	0.0102	0.0012	2.60E-18	77	-0.0058	0.0024	0.0174	0.78
**TH**	frontalpole	rs3776864	A	C	A	C	0.0057	0.0010	1.70E-08	32	-0.0028	0.0022	0.2162	0.67
**TH**	frontalpole	rs4585442	A	G	A	G	-0.0063	0.0010	8.10E-10	38	0.0051	0.0022	0.0196	0.69
**TH**	frontalpole	rs5757675	G	T	G	T	0.0065	0.0011	2.70E-09	34	-0.0001	0.0023	0.9823	0.26
**TH**	frontalpole	rs59779556	T	G	T	G	0.0055	0.0010	2.00E-08	32	-0.0004	0.0020	0.8439	0.55
**TH**	frontalpole	rs7524118	T	C	T	C	-0.0058	0.0011	4.90E-08	30	0.0011	0.0022	0.6087	0.29
**TH**	frontalpole	rs9321171	C	T	C	T	0.0054	0.0010	4.20E-08	31	0.0024	0.0020	0.2420	0.54
**SA**	inferiorparietal	rs11763750	G	A	G	A	0.0072	0.0012	5.10E-09	34	2.9415	4.7741	0.5378	0.81
**SA**	inferiorparietal	rs1229762	C	T	C	T	-0.0072	0.0010	1.00E-12	51	8.7219	4.1375	0.0350	0.34

**SA**	inferiorparietal	rs12518468	T	C	T	C	-0.0059	0.0010	8.50E-09	33	2.0346	4.2059	0.6286	0.67
**SA**	inferiorparietal	rs12567114	G	A	G	A	0.0063	0.0011	4.10E-09	34	-1.8447	4.4771	0.6803	0.72
**SA**	inferiorparietal	rs12661667	C	T	C	T	-0.0060	0.0011	2.80E-08	31	2.1440	4.3749	0.6241	0.74
**SA**	inferiorparietal	rs13107325	C	T	C	T	-0.0133	0.0018	2.50E-13	53	20.8837	8.0238	0.0092	0.93
**SA**	inferiorparietal	rs1380703	A	G	A	G	-0.0068	0.0010	1.60E-11	45	-3.7113	4.2039	0.3773	0.62
**SA**	inferiorparietal	rs1607227	G	T	G	T	0.0064	0.0011	1.50E-09	36	-4.0498	4.3661	0.3536	0.70
**SA**	inferiorparietal	rs17005118	G	A	G	A	-0.0065	0.0011	2.50E-09	36	1.6572	4.3513	0.7033	0.74
**SA**	inferiorparietal	rs17388803	A	C	A	C	-0.0098	0.0016	6.50E-10	38	-1.9416	6.2492	0.7560	0.89
**SA**	inferiorparietal	rs2014830	C	T	C	T	0.0058	0.0011	2.70E-08	30	-8.8896	4.2629	0.0370	0.70
**SA**	inferiorparietal	rs205024	C	T	C	T	0.0055	0.0010	2.70E-08	31	-0.6230	3.8823	0.8725	0.62
**SA**	inferiorparietal	rs2820313	A	G	A	G	-0.0060	0.0010	2.30E-09	35	-0.5026	4.0569	0.9014	0.66
**SA**	inferiorparietal	rs2863957	C	A	C	A	0.0102	0.0012	2.60E-18	77	-3.7439	4.7266	0.4283	0.78
**SA**	inferiorparietal	rs3776864	A	C	A	C	0.0057	0.0010	1.70E-08	32	3.4046	4.3045	0.4290	0.67
**SA**	inferiorparietal	rs4585442	A	G	A	G	-0.0063	0.0010	8.10E-10	38	0.9440	4.2129	0.8227	0.69
**SA**	inferiorparietal	rs5757675	G	T	G	T	0.0065	0.0011	2.70E-09	34	-4.1812	4.3906	0.3409	0.26
**SA**	inferiorparietal	rs59779556	T	G	T	G	0.0055	0.0010	2.00E-08	32	-4.1927	3.8654	0.2781	0.55
**SA**	inferiorparietal	rs7524118	T	C	T	C	-0.0058	0.0011	4.90E-08	30	-0.5696	4.2115	0.8924	0.29
**SA**	inferiorparietal	rs9321171	C	T	C	T	0.0054	0.0010	4.20E-08	31	1.3536	3.9127	0.7294	0.54
**SA**	lateraloccipital	rs11763750	G	A	G	A	0.0072	0.0012	5.10E-09	34	-1.3403	4.2208	0.7508	0.81
**SA**	lateraloccipital	rs1229762	C	T	C	T	-0.0072	0.0010	1.00E-12	51	-3.4818	3.6502	0.3402	0.34
**SA**	lateraloccipital	rs12518468	T	C	T	C	-0.0059	0.0010	8.50E-09	33	2.6735	3.7229	0.4727	0.67
**SA**	lateraloccipital	rs12661667	C	T	C	T	-0.0060	0.0011	2.80E-08	31	-5.6549	3.8692	0.1439	0.74
**SA**	lateraloccipital	rs12963463	C	T	C	T	0.0071	0.0011	1.90E-11	45	3.0734	3.8666	0.4267	0.30
**SA**	lateraloccipital	rs13107325	C	T	C	T	-0.0133	0.0018	2.50E-13	53	-8.3266	7.1156	0.2419	0.93
**SA**	lateraloccipital	rs1380703	A	G	A	G	-0.0068	0.0010	1.60E-11	45	-6.0516	3.7097	0.1028	0.62

**SA**	lateraloccipital	rs1607227	G	T	G	T	0.0064	0.0011	1.50E-09	36	8.5572	3.8564	0.0265	0.70
**SA**	lateraloccipital	rs17005118	G	A	G	A	-0.0065	0.0011	2.50E-09	36	-5.7842	3.8552	0.1335	0.74
**SA**	lateraloccipital	rs205024	C	T	C	T	0.0055	0.0010	2.70E-08	31	-2.3272	3.4286	0.4973	0.62
**SA**	lateraloccipital	rs2820313	A	G	A	G	-0.0060	0.0010	2.30E-09	35	3.5288	3.5863	0.3251	0.66
**SA**	lateraloccipital	rs2863957	C	A	C	A	0.0102	0.0012	2.60E-18	77	2.2826	4.1749	0.5846	0.78
**SA**	lateraloccipital	rs3776864	A	C	A	C	0.0057	0.0010	1.70E-08	32	7.1127	3.8129	0.0621	0.67
**SA**	lateraloccipital	rs4585442	A	G	A	G	-0.0063	0.0010	8.10E-10	38	-7.5930	3.7285	0.0417	0.69
**SA**	lateraloccipital	rs5757675	G	T	G	T	0.0065	0.0011	2.70E-09	34	0.3999	3.8763	0.9178	0.26
**SA**	lateraloccipital	rs7524118	T	C	T	C	-0.0058	0.0011	4.90E-08	30	2.3285	3.7311	0.5326	0.29
**SA**	lateraloccipital	rs9321171	C	T	C	T	0.0054	0.0010	4.20E-08	31	2.1689	3.4569	0.5304	0.54
**SA**	middletemporal	rs11763750	G	A	G	A	0.0072	0.1705	5.10E-09	34	0.1705	2.7406	0.9504	0.81
**SA**	middletemporal	rs12518468	T	C	T	C	-0.0059	-3.1308	8.50E-09	33	-3.1308	2.4134	0.1945	0.67
**SA**	middletemporal	rs12567114	G	A	G	A	0.0063	-0.6639	4.10E-09	34	-0.6639	2.5656	0.7958	0.72
**SA**	middletemporal	rs12661667	C	T	C	T	-0.0060	-1.7562	2.80E-08	31	-1.7562	2.5216	0.4861	0.74
**SA**	middletemporal	rs1380703	A	G	A	G	-0.0068	-3.6311	1.60E-11	45	-3.6311	2.3988	0.1301	0.62
**SA**	middletemporal	rs1607227	G	T	G	T	0.0064	1.9918	1.50E-09	36	1.9918	2.5037	0.4263	0.70
**SA**	middletemporal	rs17005118	G	A	G	A	-0.0065	-1.1678	2.50E-09	36	-1.1678	2.5013	0.6406	0.74
**SA**	middletemporal	rs17388803	A	C	A	C	-0.0098	-1.9838	6.50E-10	38	-1.9838	3.5725	0.5787	0.89
**SA**	middletemporal	rs205024	C	T	C	T	0.0055	1.3856	2.70E-08	31	1.3856	2.2292	0.5342	0.62
**SA**	middletemporal	rs2820313	A	G	A	G	-0.0060	-3.7174	2.30E-09	35	-3.7174	2.3275	0.1102	0.66
**SA**	middletemporal	rs2863957	C	A	C	A	0.0102	3.1638	2.60E-18	77	3.1638	2.7177	0.2444	0.78
**SA**	middletemporal	rs3776864	A	C	A	C	0.0057	2.4144	1.70E-08	32	2.4144	2.4742	0.3291	0.67
**SA**	middletemporal	rs5757675	G	T	G	T	0.0065	2.5131	2.70E-09	34	2.5131	2.5232	0.3192	0.26
**SA**	middletemporal	rs59779556	T	G	T	G	0.0055	-1.0092	2.00E-08	32	-1.0092	2.2156	0.6488	0.55
**SA**	middletemporal	rs7524118	T	C	T	C	-0.0058	0.4566	4.90E-08	30	0.4566	2.4198	0.8503	0.29

**SA**	middletemporal	rs9321171	C	T	C	T	0.0054	-4.3881	4.20E-08	31	-4.3881	2.2382	0.0499	0.54
**TH**	middletemporal	rs11763750	G	A	G	A	0.0072	0.0005	5.10E-09	34	0.0005	0.0012	0.6692	0.81
**TH**	middletemporal	rs1229762	C	T	C	T	-0.0072	-0.0013	1.00E-12	51	-0.0013	0.0010	0.2066	0.34
**TH**	middletemporal	rs12518468	T	C	T	C	-0.0059	0.0010	8.50E-09	33	0.0010	0.0010	0.3226	0.67
**TH**	middletemporal	rs12661667	C	T	C	T	-0.0060	-0.0022	2.80E-08	31	-0.0022	0.0011	0.0369	0.74
**TH**	middletemporal	rs13107325	C	T	C	T	-0.0133	-0.0047	2.50E-13	53	-0.0047	0.0020	0.0154	0.93
**TH**	middletemporal	rs1380703	A	G	A	G	-0.0068	-0.0003	1.60E-11	45	-0.0003	0.0010	0.7776	0.62
**TH**	middletemporal	rs1607227	G	T	G	T	0.0064	0.0010	1.50E-09	36	0.0010	0.0011	0.3628	0.70
**TH**	middletemporal	rs17005118	G	A	G	A	-0.0065	-0.0017	2.50E-09	36	-0.0017	0.0011	0.1101	0.74
**TH**	middletemporal	rs17388803	A	C	A	C	-0.0098	-0.0012	6.50E-10	38	-0.0012	0.0015	0.4118	0.89
**TH**	middletemporal	rs2014830	C	T	C	T	0.0058	0.0006	2.70E-08	30	0.0006	0.0010	0.5676	0.70
**TH**	middletemporal	rs205024	C	T	C	T	0.0055	0.0013	2.70E-08	31	0.0013	0.0009	0.1553	0.62
**TH**	middletemporal	rs2820313	A	G	A	G	-0.0060	-0.0011	2.30E-09	35	-0.0011	0.0010	0.2768	0.66
**TH**	middletemporal	rs2863957	C	A	C	A	0.0102	0.0001	2.60E-18	77	0.0001	0.0012	0.9242	0.78
**TH**	middletemporal	rs3776864	A	C	A	C	0.0057	-0.0012	1.70E-08	32	-0.0012	0.0011	0.2500	0.67
**TH**	middletemporal	rs5757675	G	T	G	T	0.0065	0.0012	2.70E-09	34	0.0012	0.0011	0.2545	0.26
**TH**	middletemporal	rs59779556	T	G	T	G	0.0055	-0.0004	2.00E-08	32	-0.0004	0.0009	0.6393	0.55
**TH**	middletemporal	rs7524118	T	C	T	C	-0.0058	-0.0015	4.90E-08	30	-0.0015	0.0010	0.1492	0.29
**TH**	paracentral	rs11763750	G	A	G	A	0.0072	0.0012	5.10E-09	34	-0.0002	0.0012	0.8760	0.81
**TH**	paracentral	rs1229762	C	T	C	T	-0.0072	0.0010	1.00E-12	51	0.0024	0.0010	0.0200	0.34
**TH**	paracentral	rs12518468	T	C	T	C	-0.0059	0.0010	8.50E-09	33	-0.0003	0.0010	0.7953	0.67
**TH**	paracentral	rs12661667	C	T	C	T	-0.0060	0.0011	2.80E-08	31	-0.0007	0.0011	0.5225	0.74
**TH**	paracentral	rs12963463	C	T	C	T	0.0071	0.0011	1.90E-11	45	-0.0014	0.0011	0.1951	0.30
**TH**	paracentral	rs1380703	A	G	A	G	-0.0068	0.0010	1.60E-11	45	0.0002	0.0011	0.8800	0.62
**TH**	paracentral	rs17005118	G	A	G	A	-0.0065	0.0011	2.50E-09	36	0.0016	0.0011	0.1414	0.74

**TH**	paracentral	rs17388803	A	C	A	C	-0.0098	0.0016	6.50E-10	38	0.0024	0.0016	0.1302	0.89
**TH**	paracentral	rs2014830	C	T	C	T	0.0058	0.0011	2.70E-08	30	-0.0009	0.0011	0.4037	0.70
**TH**	paracentral	rs205024	C	T	C	T	0.0055	0.0010	2.70E-08	31	-0.0011	0.0010	0.2463	0.62
**TH**	paracentral	rs2820313	A	G	A	G	-0.0060	0.0010	2.30E-09	35	0.0002	0.0010	0.8403	0.66
**TH**	paracentral	rs2863957	C	A	C	A	0.0102	0.0012	2.60E-18	77	0.0005	0.0012	0.6944	0.78
**TH**	paracentral	rs3776864	A	C	A	C	0.0057	0.0010	1.70E-08	32	0.0006	0.0011	0.5911	0.67
**TH**	paracentral	rs4585442	A	G	A	G	-0.0063	0.0010	8.10E-10	38	0.0015	0.0011	0.1543	0.69
**TH**	paracentral	rs59779556	T	G	T	G	0.0055	0.0010	2.00E-08	32	-0.0001	0.0010	0.9044	0.55
**TH**	paracentral	rs7524118	T	C	T	C	-0.0058	0.0011	4.90E-08	30	0.0013	0.0011	0.2113	0.29
**TH**	paracentral	rs9321171	C	T	C	T	0.0054	0.0010	4.20E-08	31	-0.0015	0.0010	0.1168	0.54
**TH**	parahippocampal	rs11763750	G	A	G	A	0.0072	0.0012	5.10E-09	34	-0.0021	0.0029	0.4736	34.18
**TH**	parahippocampal	rs1229762	C	T	C	T	-0.0072	0.0010	1.00E-12	51	0.0003	0.0025	0.9144	50.71
**TH**	parahippocampal	rs12518468	T	C	T	C	-0.0059	0.0010	8.50E-09	33	-0.0002	0.0026	0.9362	33.22
**TH**	parahippocampal	rs12567114	G	A	G	A	0.0063	0.0011	4.10E-09	34	-0.0046	0.0027	0.0879	34.50
**TH**	parahippocampal	rs12661667	C	T	C	T	-0.0060	0.0011	2.80E-08	31	0.0014	0.0027	0.5878	30.68
**TH**	parahippocampal	rs13107325	C	T	C	T	-0.0133	0.0018	2.50E-13	53	0.0059	0.0049	0.2293	52.67
**TH**	parahippocampal	rs1380703	A	G	A	G	-0.0068	0.0010	1.60E-11	45	0.0013	0.0026	0.6218	45.34
**TH**	parahippocampal	rs1607227	G	T	G	T	0.0064	0.0011	1.50E-09	36	-0.0010	0.0027	0.7097	36.47
**TH**	parahippocampal	rs17005118	G	A	G	A	-0.0065	0.0011	2.50E-09	36	0.0021	0.0027	0.4422	35.56
**TH**	parahippocampal	rs17388803	A	C	A	C	-0.0098	0.0016	6.50E-10	38	0.0003	0.0038	0.9296	38.33
**TH**	parahippocampal	rs2014830	C	T	C	T	0.0058	0.0011	2.70E-08	30	-0.0020	0.0026	0.4339	30.35
**TH**	parahippocampal	rs205024	C	T	C	T	0.0055	0.0010	2.70E-08	31	-0.0003	0.0024	0.8924	31.23
**TH**	parahippocampal	rs2820313	A	G	A	G	-0.0060	0.0010	2.30E-09	35	0.0036	0.0025	0.1498	35.35
**TH**	parahippocampal	rs2863957	C	A	C	A	0.0102	0.0012	2.60E-18	77	-0.0009	0.0029	0.7477	77.04
**TH**	parahippocampal	rs3776864	A	C	A	C	0.0057	0.0010	1.70E-08	32	0.0041	0.0026	0.1182	31.54

**TH**	parahippocampal	rs4585442	A	G	A	G	-0.0063	0.0010	8.10E-10	38	-0.0001	0.0026	0.9594	37.51
**TH**	parahippocampal	rs5757675	G	T	G	T	0.0065	0.0011	2.70E-09	34	-0.0065	0.0027	0.0150	34.50
**TH**	parahippocampal	rs59779556	T	G	T	G	0.0055	0.0010	2.00E-08	32	-0.0015	0.0024	0.5272	32.32
**TH**	parahippocampal	rs7524118	T	C	T	C	-0.0058	0.0011	4.90E-08	30	0.0020	0.0026	0.4335	29.90
**TH**	parahippocampal	rs9321171	C	T	C	T	0.0054	0.0010	4.20E-08	31	-0.0040	0.0024	0.0985	30.72
**TH**	superiortemporal	rs11763750	G	A	G	A	0.0072	0.0010	5.10E-09	34	0.0010	0.0012	0.3688	0.81
**TH**	superiortemporal	rs1229762	C	T	C	T	-0.0072	-0.0012	1.00E-12	51	-0.0012	0.0010	0.2168	0.34
**TH**	superiortemporal	rs12518468	T	C	T	C	-0.0059	0.0017	8.50E-09	33	0.0017	0.0010	0.0974	0.67
**TH**	superiortemporal	rs12567114	G	A	G	A	0.0063	-0.0009	4.10E-09	34	-0.0009	0.0011	0.4238	0.72
**TH**	superiortemporal	rs12661667	C	T	C	T	-0.0060	-0.0014	2.80E-08	31	-0.0014	0.0011	0.1889	0.74
**TH**	superiortemporal	rs13107325	C	T	C	T	-0.0133	-0.0040	2.50E-13	53	-0.0040	0.0018	0.0410	0.93
**TH**	superiortemporal	rs1380703	A	G	A	G	-0.0068	-0.0012	1.60E-11	45	-0.0012	0.0010	0.2396	0.62
**TH**	superiortemporal	rs1607227	G	T	G	T	0.0064	0.0019	1.50E-09	36	0.0019	0.0011	0.0699	0.70
**TH**	superiortemporal	rs17005118	G	A	G	A	-0.0065	0.0001	2.50E-09	36	0.0001	0.0011	0.9355	0.74
**TH**	superiortemporal	rs17388803	A	C	A	C	-0.0098	-0.0021	6.50E-10	38	-0.0021	0.0016	0.1553	0.89
**TH**	superiortemporal	rs2014830	C	T	C	T	0.0058	0.0010	2.70E-08	30	0.0010	0.0011	0.3468	0.70
**TH**	superiortemporal	rs205024	C	T	C	T	0.0055	-0.0010	2.70E-08	31	-0.0010	0.0010	0.2744	0.62
**TH**	superiortemporal	rs2820313	A	G	A	G	-0.0060	-0.0009	2.30E-09	35	-0.0009	0.0010	0.3399	0.66
**TH**	superiortemporal	rs2863957	C	A	C	A	0.0102	-0.0005	2.60E-18	77	-0.0005	0.0012	0.6417	0.78
**TH**	superiortemporal	rs3776864	A	C	A	C	0.0057	0.0011	1.70E-08	32	0.0011	0.0010	0.3053	0.67
**TH**	superiortemporal	rs4585442	A	G	A	G	-0.0063	-0.0010	8.10E-10	38	-0.0010	0.0010	0.3214	0.69
**TH**	superiortemporal	rs5757675	G	T	G	T	0.0065	0.0006	2.70E-09	34	0.0006	0.0011	0.6007	0.26
**TH**	superiortemporal	rs59779556	T	G	T	G	0.0055	0.0007	2.00E-08	32	0.0007	0.0010	0.4321	0.55
**TH**	superiortemporal	rs7524118	T	C	T	C	-0.0058	0.0005	4.90E-08	30	0.0005	0.0011	0.6406	0.29
**TH**	superiortemporal	rs9321171	C	T	C	T	0.0054	0.0013	4.20E-08	31	0.0013	0.0010	0.1751	0.54

Table S1. The details of genetic instruments predicting short sleep and cortical SA/TH

The details of genetic instruments predicting morningness and cortical SA/TH														
cortical SA/TH	Exposure	SNP	EA.exposure	OA.exposure	EA.outcome	OA.outcome	beta.exposure	se.exposure	pval.exposure	F	beta.outcome	se.outcome	pval.outcome	MAF
SA	cuneus	rs10518440	G	A	G	A	0.0310	0.0030	2.07E-24	28	-0.8586	1.7885	0.6312	0.16
SA	cuneus	rs10737452	C	T	C	T	0.0159	0.0023	6.30E-12	22	1.0576	1.3646	0.4383	0.38
SA	cuneus	rs1109088	G	A	G	A	-0.0129	0.0023	2.09E-08	15	0.1738	1.3766	0.8995	0.43
SA	cuneus	rs112439757	T	C	T	C	-0.0219	0.0029	6.57E-14	17	-2.1498	1.7215	0.2117	0.18
SA	cuneus	rs12054920	T	C	T	C	-0.0156	0.0022	3.95E-12	24	0.0857	1.3355	0.9488	0.48
SA	cuneus	rs12206116	A	G	A	G	0.0126	0.0023	4.40E-08	14	3.0260	1.3496	0.0250	0.40
SA	cuneus	rs12431576	C	T	C	T	-0.0148	0.0024	7.63E-10	17	-2.1763	1.4281	0.1275	0.33
SA	cuneus	rs12927162	G	A	G	A	-0.0234	0.0025	9.89E-21	35	0.5742	1.6515	0.7281	0.28
SA	cuneus	rs12956276	A	G	A	G	0.0132	0.0023	1.45E-08	15	1.4471	1.4123	0.3055	0.35

SA	cuneus	rs13167796	A	G	A	G	-0.0147	0.0026	1.67E-08	12	-2.1946	1.5465	0.1559	0.25
SA	cuneus	rs1323591	T	C	T	C	-0.0143	0.0024	1.64E-09	16	-1.5007	1.4470	0.2997	0.32
SA	cuneus	rs139911	C	T	C	T	0.0183	0.0023	9.94E-16	31	0.1495	1.3512	0.9119	0.42
SA	cuneus	rs1421085	C	T	C	T	0.0215	0.0023	4.08E-21	43	0.7427	1.3397	0.5793	0.40
SA	cuneus	rs17448682	T	C	T	C	0.0160	0.0027	1.75E-09	13	0.7654	1.5751	0.6270	0.23
SA	cuneus	rs17697212	C	T	C	T	-0.0162	0.0028	1.02E-08	10	-0.7975	1.6610	0.6311	0.20
SA	cuneus	rs202157	C	T	C	T	0.0193	0.0025	4.42E-15	26	1.0153	1.4384	0.4803	0.30
SA	cuneus	rs2232839	T	C	T	C	-0.0211	0.0028	4.81E-14	19	0.5419	1.8221	0.7662	0.21
SA	cuneus	rs2291589	G	T	G	T	-0.0165	0.0023	8.13E-13	24	-2.3538	1.3816	0.0884	0.38
SA	cuneus	rs2653349	A	G	A	G	0.0295	0.0027	4.50E-27	39	0.3027	1.6395	0.8535	0.21
SA	cuneus	rs2701524	C	T	C	T	-0.0126	0.0023	4.36E-08	15	0.6953	1.4043	0.6205	0.41
SA	cuneus	rs2794682	T	C	T	C	0.0170	0.0023	9.34E-14	27	2.4473	1.3658	0.0732	0.40
SA	cuneus	rs28634184	T	C	T	C	-0.0142	0.0026	3.97E-08	11	-0.1421	1.5422	0.9266	0.25
SA	cuneus	rs2881955	T	C	T	C	0.0144	0.0025	8.59E-09	13	2.1968	1.4724	0.1357	0.28
SA	cuneus	rs2916142	T	C	T	C	0.0135	0.0023	4.41E-09	17	2.8526	1.3349	0.0326	0.46
SA	cuneus	rs2971970	T	G	T	G	0.0168	0.0027	6.19E-10	13	-0.4235	1.6250	0.7944	0.22
SA	cuneus	rs3100052	A	G	A	G	0.0126	0.0023	4.35E-08	14	-0.2379	1.3740	0.8625	0.39
SA	cuneus	rs34571177	G	T	G	T	-0.0174	0.0029	2.68E-09	10	-0.6449	1.7256	0.7086	0.18
SA	cuneus	rs35376824	G	A	G	A	-0.0161	0.0026	1.12E-09	13	-1.0022	1.5760	0.5249	0.23
SA	cuneus	rs35935113	C	T	C	T	-0.0124	0.0023	3.59E-08	15	0.6408	1.3216	0.6278	0.45
SA	cuneus	rs36117029	G	A	G	A	0.0129	0.0024	3.76E-08	14	0.4389	1.3871	0.7517	0.36
SA	cuneus	rs3808477	T	C	T	C	0.0174	0.0025	3.30E-12	20	-0.8420	1.4576	0.5635	0.28
SA	cuneus	rs4241964	G	T	G	T	0.0160	0.0023	1.41E-12	25	0.2869	1.3648	0.8335	0.48
SA	cuneus	rs4245555	C	T	C	T	0.0173	0.0023	6.13E-14	27	2.9802	1.3438	0.0266	0.41
SA	cuneus	rs4549082	C	T	C	T	0.0170	0.0022	3.61E-14	29	0.7706	1.4341	0.5911	0.48

SA	cuneus	rs4550384	G	T	G	T	-0.0146	0.0026	2.49E-08	11	1.8023	1.5495	0.2448	0.24
SA	cuneus	rs4588790	T	C	T	C	-0.0130	0.0023	1.84E-08	15	-0.3081	1.3435	0.8186	0.38
SA	cuneus	rs4622900	C	T	C	T	-0.0125	0.0023	2.99E-08	15	-0.9805	1.3319	0.4616	0.44
SA	cuneus	rs4899502	G	A	G	A	0.0136	0.0024	1.83E-08	14	-0.5164	1.4208	0.7162	0.31
SA	cuneus	rs4936289	G	A	G	A	0.0131	0.0023	1.33E-08	15	2.2864	1.3967	0.1016	0.38
SA	cuneus	rs55910701	T	C	T	C	-0.0179	0.0026	1.05E-11	17	1.6736	1.5594	0.2832	0.24
SA	cuneus	rs56049037	A	G	A	G	-0.0159	0.0025	1.64E-10	17	1.0664	1.5813	0.5001	0.29
SA	cuneus	rs56076457	C	T	C	T	-0.0127	0.0022	1.58E-08	16	-0.0858	1.3202	0.9482	0.47
SA	cuneus	rs6131942	A	G	A	G	-0.0136	0.0023	2.31E-09	17	-0.7285	1.3397	0.5866	0.42
SA	cuneus	rs61773390	T	G	T	G	0.0258	0.0028	5.34E-20	26	3.3037	1.6767	0.0488	0.20
SA	cuneus	rs62172117	A	G	A	G	-0.0159	0.0023	1.08E-11	21	0.9887	1.3801	0.4737	0.36
SA	cuneus	rs62182115	T	C	T	C	-0.0135	0.0024	1.04E-08	15	1.0768	1.4437	0.4558	0.34
SA	cuneus	rs6441371	A	C	A	C	0.0140	0.0023	5.54E-10	19	0.6157	1.3071	0.6376	0.48
SA	cuneus	rs6778003	T	G	T	G	0.0152	0.0025	6.05E-10	16	-0.0115	1.5648	0.9941	0.29
SA	cuneus	rs6990077	T	C	T	C	-0.0185	0.0027	7.90E-12	16	-1.0922	1.6128	0.4983	0.22
SA	cuneus	rs7196720	T	C	T	C	0.0125	0.0022	2.23E-08	16	2.9308	1.3428	0.0291	0.49
SA	cuneus	rs72720396	G	A	G	A	0.0223	0.0027	4.36E-17	25	2.0885	1.6312	0.2004	0.23
SA	cuneus	rs7304278	A	G	A	G	-0.0149	0.0025	3.59E-09	14	2.1070	1.4593	0.1488	0.28
SA	cuneus	rs73095857	T	G	T	G	0.0134	0.0023	3.52E-09	17	-1.7824	1.3928	0.2006	0.42
SA	cuneus	rs7313852	A	G	A	G	-0.0226	0.0023	2.52E-23	49	-1.2072	1.3777	0.3809	0.44
SA	cuneus	rs735459	C	T	C	T	0.0155	0.0024	5.25E-11	20	-0.1166	1.4021	0.9337	0.36
SA	cuneus	rs7598252	G	A	G	A	-0.0128	0.0023	2.00E-08	16	0.1099	1.3243	0.9339	0.47
SA	cuneus	rs7783012	G	A	G	A	-0.0140	0.0023	1.09E-09	18	-3.5002	1.3418	0.0091	0.41
SA	cuneus	rs77960	A	G	A	G	0.0136	0.0024	1.22E-08	14	-1.3926	1.4236	0.3279	0.33
SA	cuneus	rs7925560	A	G	A	G	-0.0173	0.0027	6.54E-11	15	-0.7785	1.5421	0.6137	0.23

**SA**	**cuneus**	**rs7959983**	**C**	**T**	**C**	**T**	**0.0151**	**0.0023**	**3.17E-11**	**21**	**-0.3672**	**1.3461**	**0.7850**	**0.40**
**SA**	**cuneus**	**rs8072507**	**C**	**T**	**C**	**T**	**0.0150**	**0.0025**	**1.37E-09**	**15**	**-3.2320**	**1.4316**	**0.0240**	**0.29**
**SA**	**cuneus**	**rs9348050**	**T**	**C**	**T**	**C**	**0.0133**	**0.0022**	**3.44E-09**	**17**	**-0.2167**	**1.3376**	**0.8713**	**0.49**
**SA**	**cuneus**	**rs9395520**	**T**	**C**	**T**	**C**	**0.0180**	**0.0024**	**1.59E-13**	**23**	**0.5959**	**1.4215**	**0.6751**	**0.30**
**SA**	**cuneus**	**rs9597250**	**A**	**C**	**A**	**C**	**-0.0178**	**0.0029**	**6.12E-10**	**12**	**2.2227**	**1.6590**	**0.1803**	**0.19**
**SA**	**cuneus**	**rs974552**	**G**	**A**	**G**	**A**	**0.0192**	**0.0028**	**1.48E-11**	**14**	**4.8243**	**1.7686**	**0.0064**	**0.19**
**SA**	**cuneus**	**rs9795439**	**A**	**G**	**A**	**G**	**0.0163**	**0.0028**	**6.68E-09**	**11**	**1.0536**	**1.9343**	**0.5859**	**0.20**
**SA**	**cuneus**	**rs9964420**	**A**	**C**	**A**	**C**	**-0.0202**	**0.0025**	**2.46E-16**	**28**	**1.0271**	**1.4635**	**0.4828**	**0.30**
**SA**	**inferiorparietal**	**rs10518440**	**G**	**A**	**G**	**A**	**0.0310**	**0.0030**	**2.07E-24**	**28**	**-2.6100**	**5.1952**	**0.6154**	**0.16**
**SA**	**inferiorparietal**	**rs10737452**	**C**	**T**	**C**	**T**	**0.0159**	**0.0023**	**6.30E-12**	**22**	**-4.4198**	**3.9687**	**0.2654**	**0.38**
**SA**	**inferiorparietal**	**rs112439757**	**T**	**C**	**T**	**C**	**-0.0219**	**0.0029**	**6.57E-14**	**17**	**-7.4379**	**5.0071**	**0.1374**	**0.18**
**SA**	**inferiorparietal**	**rs12206116**	**A**	**G**	**A**	**G**	**0.0126**	**0.0023**	**4.40E-08**	**14**	**-6.4759**	**3.9348**	**0.0998**	**0.40**
**SA**	**inferiorparietal**	**rs12431576**	**C**	**T**	**C**	**T**	**-0.0148**	**0.0024**	**7.63E-10**	**17**	**6.1051**	**4.1540**	**0.1416**	**0.33**
**SA**	**inferiorparietal**	**rs12927162**	**G**	**A**	**G**	**A**	**-0.0234**	**0.0025**	**9.89E-21**	**35**	**0.5880**	**4.8092**	**0.9027**	**0.28**
**SA**	**inferiorparietal**	**rs12956276**	**A**	**G**	**A**	**G**	**0.0132**	**0.0023**	**1.45E-08**	**15**	**2.8852**	**4.1130**	**0.4830**	**0.35**
**SA**	**inferiorparietal**	**rs1323591**	**T**	**C**	**T**	**C**	**-0.0143**	**0.0024**	**1.64E-09**	**16**	**3.4054**	**4.2180**	**0.4195**	**0.32**
**SA**	**inferiorparietal**	**rs139911**	**C**	**T**	**C**	**T**	**0.0183**	**0.0023**	**9.94E-16**	**31**	**-7.1841**	**3.9276**	**0.0674**	**0.42**

**SA**	**inferiorparietal**	**rs1421085**	**C**	**T**	**C**	**T**	**0.0215**	**0.0023**	**4.08E-21**	**43**	**-3.3393**	**3.8935**	**0.3911**	**0.40**
**SA**	**inferiorparietal**	**rs17448682**	**T**	**C**	**T**	**C**	**0.0160**	**0.0027**	**1.75E-09**	**13**	**-6.3976**	**4.5929**	**0.1636**	**0.23**
**SA**	**inferiorparietal**	**rs17697212**	**C**	**T**	**C**	**T**	**-0.0162**	**0.0028**	**1.02E-08**	**10**	**7.7073**	**4.8279**	**0.1104**	**0.20**
**SA**	**inferiorparietal**	**rs202157**	**C**	**T**	**C**	**T**	**0.0193**	**0.0025**	**4.42E-15**	**26**	**-3.3108**	**4.1861**	**0.4290**	**0.30**
**SA**	**inferiorparietal**	**rs2232839**	**T**	**C**	**T**	**C**	**-0.0211**	**0.0028**	**4.81E-14**	**19**	**1.0715**	**5.3348**	**0.8408**	**0.21**
**SA**	**inferiorparietal**	**rs2291589**	**G**	**T**	**G**	**T**	**-0.0165**	**0.0023**	**8.13E-13**	**24**	**2.6289**	**4.0260**	**0.5138**	**0.38**
**SA**	**inferiorparietal**	**rs2653349**	**A**	**G**	**A**	**G**	**0.0295**	**0.0027**	**4.50E-27**	**39**	**5.8314**	**4.7748**	**0.2220**	**0.21**
**SA**	**inferiorparietal**	**rs2701524**	**C**	**T**	**C**	**T**	**-0.0126**	**0.0023**	**4.36E-08**	**15**	**6.1712**	**4.0960**	**0.1319**	**0.41**
**SA**	**inferiorparietal**	**rs2794682**	**T**	**C**	**T**	**C**	**0.0170**	**0.0023**	**9.34E-14**	**27**	**0.1355**	**3.9653**	**0.9727**	**0.40**
**SA**	**inferiorparietal**	**rs2881955**	**T**	**C**	**T**	**C**	**0.0144**	**0.0025**	**8.59E-09**	**13**	**0.3045**	**4.2728**	**0.9432**	**0.28**
**SA**	**inferiorparietal**	**rs2916142**	**T**	**C**	**T**	**C**	**0.0135**	**0.0023**	**4.41E-09**	**17**	**0.2249**	**3.8771**	**0.9537**	**0.46**
**SA**	**inferiorparietal**	**rs3100052**	**A**	**G**	**A**	**G**	**0.0126**	**0.0023**	**4.35E-08**	**14**	**-1.6799**	**4.0024**	**0.6747**	**0.39**
**SA**	**inferiorparietal**	**rs34571177**	**G**	**T**	**G**	**T**	**-0.0174**	**0.0029**	**2.68E-09**	**10**	**2.3556**	**5.0307**	**0.6396**	**0.18**

**SA**	**inferiorparietal**	**rs35376824**	**G**	**A**	**G**	**A**	**-0.0161**	**0.0026**	**1.12E-09**	**13**	**4.5813**	**4.5959**	**0.3188**	**0.23**
**SA**	**inferiorparietal**	**rs35935113**	**C**	**T**	**C**	**T**	**-0.0124**	**0.0023**	**3.59E-08**	**15**	**2.7217**	**3.8466**	**0.4792**	**0.45**
**SA**	**inferiorparietal**	**rs36117029**	**G**	**A**	**G**	**A**	**0.0129**	**0.0024**	**3.76E-08**	**14**	**-0.8722**	**4.0352**	**0.8289**	**0.36**
**SA**	**inferiorparietal**	**rs3808477**	**T**	**C**	**T**	**C**	**0.0174**	**0.0025**	**3.30E-12**	**20**	**-1.7137**	**4.2453**	**0.6865**	**0.28**
**SA**	**inferiorparietal**	**rs4241964**	**G**	**T**	**G**	**T**	**0.0160**	**0.0023**	**1.41E-12**	**25**	**3.4298**	**3.9686**	**0.3875**	**0.48**
**SA**	**inferiorparietal**	**rs4245555**	**C**	**T**	**C**	**T**	**0.0173**	**0.0023**	**6.13E-14**	**27**	**-5.0701**	**3.9029**	**0.1939**	**0.41**
**SA**	**inferiorparietal**	**rs4549082**	**C**	**T**	**C**	**T**	**0.0170**	**0.0022**	**3.61E-14**	**29**	**0.0768**	**4.1564**	**0.9853**	**0.48**
**SA**	**inferiorparietal**	**rs4550384**	**G**	**T**	**G**	**T**	**-0.0146**	**0.0026**	**2.49E-08**	**11**	**4.1377**	**4.5226**	**0.3603**	**0.24**
**SA**	**inferiorparietal**	**rs4588790**	**T**	**C**	**T**	**C**	**-0.0130**	**0.0023**	**1.84E-08**	**15**	**3.7418**	**3.9159**	**0.3393**	**0.38**
**SA**	**inferiorparietal**	**rs4622900**	**C**	**T**	**C**	**T**	**-0.0125**	**0.0023**	**2.99E-08**	**15**	**-2.6864**	**3.8717**	**0.4878**	**0.44**
**SA**	**inferiorparietal**	**rs4899502**	**G**	**A**	**G**	**A**	**0.0136**	**0.0024**	**1.83E-08**	**14**	**-1.3011**	**4.1336**	**0.7529**	**0.31**
**SA**	**inferiorparietal**	**rs4936289**	**G**	**A**	**G**	**A**	**0.0131**	**0.0023**	**1.33E-08**	**15**	**-2.6383**	**4.0544**	**0.5152**	**0.38**
**SA**	**inferiorparietal**	**rs55910701**	**T**	**C**	**T**	**C**	**-0.0179**	**0.0026**	**1.05E-11**	**17**	**-10.7108**	**4.5407**	**0.0183**	**0.24**

**SA**	**inferiorparietal**	**rs56049037**	**A**	**G**	**A**	**G**	**-0.0159**	**0.0025**	**1.64E-10**	**17**	**-3.3921**	**4.6175**	**0.4626**	**0.29**
**SA**	**inferiorparietal**	**rs56076457**	**C**	**T**	**C**	**T**	**-0.0127**	**0.0022**	**1.58E-08**	**16**	**2.7936**	**3.8447**	**0.4675**	**0.47**
**SA**	**inferiorparietal**	**rs6131942**	**A**	**G**	**A**	**G**	**-0.0136**	**0.0023**	**2.31E-09**	**17**	**-0.6404**	**3.9023**	**0.8696**	**0.42**
**SA**	**inferiorparietal**	**rs61773390**	**T**	**G**	**T**	**G**	**0.0258**	**0.0028**	**5.34E-20**	**26**	**-14.6269**	**4.8818**	**0.0027**	**0.20**
**SA**	**inferiorparietal**	**rs62172117**	**A**	**G**	**A**	**G**	**-0.0159**	**0.0023**	**1.08E-11**	**21**	**-4.9141**	**4.0202**	**0.2216**	**0.36**
**SA**	**inferiorparietal**	**rs62182115**	**T**	**C**	**T**	**C**	**-0.0135**	**0.0024**	**1.04E-08**	**15**	**-4.2537**	**4.1885**	**0.3098**	**0.34**
**SA**	**inferiorparietal**	**rs6441371**	**A**	**C**	**A**	**C**	**0.0140**	**0.0023**	**5.54E-10**	**19**	**0.2032**	**3.8019**	**0.9574**	**0.48**
**SA**	**inferiorparietal**	**rs6778003**	**T**	**G**	**T**	**G**	**0.0152**	**0.0025**	**6.05E-10**	**16**	**0.4123**	**4.5568**	**0.9279**	**0.29**
**SA**	**inferiorparietal**	**rs6990077**	**T**	**C**	**T**	**C**	**-0.0185**	**0.0027**	**7.90E-12**	**16**	**1.0198**	**4.6822**	**0.8276**	**0.22**
**SA**	**inferiorparietal**	**rs7196720**	**T**	**C**	**T**	**C**	**0.0125**	**0.0022**	**2.23E-08**	**16**	**-4.3956**	**3.9029**	**0.2601**	**0.49**
**SA**	**inferiorparietal**	**rs7304278**	**A**	**G**	**A**	**G**	**-0.0149**	**0.0025**	**3.59E-09**	**14**	**0.8339**	**4.2461**	**0.8443**	**0.28**
**SA**	**inferiorparietal**	**rs73095857**	**T**	**G**	**T**	**G**	**0.0134**	**0.0023**	**3.52E-09**	**17**	**-2.7951**	**4.0396**	**0.4890**	**0.42**
**SA**	**inferiorparietal**	**rs7313852**	**A**	**G**	**A**	**G**	**-0.0226**	**0.0023**	**2.52E-23**	**49**	**-1.3740**	**4.0117**	**0.7320**	**0.44**

**SA**	**inferiorparietal**	**rs735459**	**C**	**T**	**C**	**T**	**0.0155**	**0.0024**	**5.25E-11**	**20**	**-1.9068**	**4.0838**	**0.6406**	**0.36**
**SA**	**inferiorparietal**	**rs7598252**	**G**	**A**	**G**	**A**	**-0.0128**	**0.0023**	**2.00E-08**	**16**	**-11.3370**	**3.8526**	**0.0033**	**0.47**
**SA**	**inferiorparietal**	**rs7783012**	**G**	**A**	**G**	**A**	**-0.0140**	**0.0023**	**1.09E-09**	**18**	**8.6010**	**3.9109**	**0.0279**	**0.41**
**SA**	**inferiorparietal**	**rs77960**	**A**	**G**	**A**	**G**	**0.0136**	**0.0024**	**1.22E-08**	**14**	**0.9080**	**4.1427**	**0.8265**	**0.33**
**SA**	**inferiorparietal**	**rs8072507**	**C**	**T**	**C**	**T**	**0.0150**	**0.0025**	**1.37E-09**	**15**	**9.0804**	**4.1673**	**0.0293**	**0.29**
**SA**	**inferiorparietal**	**rs9348050**	**T**	**C**	**T**	**C**	**0.0133**	**0.0022**	**3.44E-09**	**17**	**-1.2120**	**3.9026**	**0.7561**	**0.49**
**SA**	**inferiorparietal**	**rs9395520**	**T**	**C**	**T**	**C**	**0.0180**	**0.0024**	**1.59E-13**	**23**	**1.8859**	**4.1376**	**0.6485**	**0.30**
**SA**	**inferiorparietal**	**rs9597250**	**A**	**C**	**A**	**C**	**-0.0178**	**0.0029**	**6.12E-10**	**12**	**3.1225**	**4.8325**	**0.5182**	**0.19**
**SA**	**inferiorparietal**	**rs974552**	**G**	**A**	**G**	**A**	**0.0192**	**0.0028**	**1.48E-11**	**14**	**0.9950**	**5.1566**	**0.8470**	**0.19**
**SA**	**inferiorparietal**	**rs9795439**	**A**	**G**	**A**	**G**	**0.0163**	**0.0028**	**6.68E-09**	**11**	**-7.7084**	**5.6273**	**0.1707**	**0.20**
**SA**	**inferiorparietal**	**rs9964420**	**A**	**C**	**A**	**C**	**-0.0202**	**0.0025**	**2.46E-16**	**28**	**9.8477**	**4.2614**	**0.0208**	**0.30**
**SA**	**lateraloccipital**	**rs10518440**	**G**	**A**	**G**	**A**	**0.0310**	**0.0030**	**2.07E-24**	**28**	**5.5752**	**4.5963**	**0.2251**	**0.16**
**SA**	**lateraloccipital**	**rs10737452**	**C**	**T**	**C**	**T**	**0.0159**	**0.0023**	**6.30E-12**	**22**	**-0.4574**	**3.5086**	**0.8963**	**0.38**
**SA**	**lateraloccipital**	**rs1109088**	**G**	**A**	**G**	**A**	**-0.0129**	**0.0023**	**2.09E-08**	**15**	**4.9317**	**3.5478**	**0.1645**	**0.43**
**SA**	**lateraloccipital**	**rs112439757**	**T**	**C**	**T**	**C**	**-0.0219**	**0.0029**	**6.57E-14**	**17**	**-2.5956**	**4.4303**	**0.5580**	**0.18**

SA	lateraloccipital	rs12054920	T	C	T	C	-0.0156	0.0022	3.95E-12	24	-3.5377	3.4348	0.3030	0.48
SA	lateraloccipital	rs12206116	A	G	A	G	0.0126	0.0023	4.40E-08	14	3.6678	3.4764	0.2914	0.40
SA	lateraloccipital	rs12431576	C	T	C	T	-0.0148	0.0024	7.63E-10	17	-14.1514	3.6693	0.0001	0.33
SA	lateraloccipital	rs12927162	G	A	G	A	-0.0234	0.0025	9.89E-21	35	2.2252	4.2500	0.6006	0.28
SA	lateraloccipital	rs12956276	A	G	A	G	0.0132	0.0023	1.45E-08	15	6.7041	3.6363	0.0652	0.35
SA	lateraloccipital	rs13167796	A	G	A	G	-0.0147	0.0026	1.67E-08	12	4.5336	3.9756	0.2541	0.25
SA	lateraloccipital	rs1323591	T	C	T	C	-0.0143	0.0024	1.64E-09	16	-0.2289	3.7212	0.9510	0.32
SA	lateraloccipital	rs139911	C	T	C	T	0.0183	0.0023	9.94E-16	31	-5.0542	3.4755	0.1459	0.42
SA	lateraloccipital	rs1421085	C	T	C	T	0.0215	0.0023	4.08E-21	43	-2.6429	3.4443	0.4429	0.40
SA	lateraloccipital	rs17448682	T	C	T	C	0.0160	0.0027	1.75E-09	13	5.6607	4.0659	0.1638	0.23
SA	lateraloccipital	rs17697212	C	T	C	T	-0.0162	0.0028	1.02E-08	10	0.8698	4.2762	0.8388	0.20
SA	lateraloccipital	rs202157	C	T	C	T	0.0193	0.0025	4.42E-15	26	4.7258	3.7035	0.2019	0.30
SA	lateraloccipital	rs2232839	T	C	T	C	-0.0211	0.0028	4.81E-14	19	-1.9512	4.6985	0.6779	0.21
SA	lateraloccipital	rs2291589	G	T	G	T	-0.0165	0.0023	8.13E-13	24	-2.7887	3.5577	0.4331	0.38
SA	lateraloccipital	rs2653349	A	G	A	G	0.0295	0.0027	4.50E-27	39	3.6333	4.2180	0.3890	0.21
SA	lateraloccipital	rs2701524	C	T	C	T	-0.0126	0.0023	4.36E-08	15	0.2263	3.6148	0.9501	0.41
SA	lateraloccipital	rs2794682	T	C	T	C	0.0170	0.0023	9.34E-14	27	7.7863	3.5120	0.0266	0.40
SA	lateraloccipital	rs28634184	T	C	T	C	-0.0142	0.0026	3.97E-08	11	3.4668	3.9645	0.3819	0.25
SA	lateraloccipital	rs2881955	T	C	T	C	0.0144	0.0025	8.59E-09	13	5.0350	3.7825	0.1831	0.28
SA	lateraloccipital	rs2916142	T	C	T	C	0.0135	0.0023	4.41E-09	17	3.9276	3.4280	0.2519	0.46
SA	lateraloccipital	rs2971970	T	G	T	G	0.0168	0.0027	6.19E-10	13	0.7022	4.1813	0.8666	0.22
SA	lateraloccipital	rs3100052	A	G	A	G	0.0126	0.0023	4.35E-08	14	3.1722	3.5360	0.3696	0.39
SA	lateraloccipital	rs34571177	G	T	G	T	-0.0174	0.0029	2.68E-09	10	-6.2207	4.4410	0.1613	0.18
SA	lateraloccipital	rs35376824	G	A	G	A	-0.0161	0.0026	1.12E-09	13	-1.5265	4.0595	0.7069	0.23
SA	lateraloccipital	rs35935113	C	T	C	T	-0.0124	0.0023	3.59E-08	15	-4.0243	3.4000	0.2366	0.45

**SA**	**lateraloccipital**	**rs36117029**	**G**	**A**	**G**	**A**	**0.0129**	**0.0024**	**3.76E-08**	**14**	**2.4099**	**3.5710**	**0.4998**	**0.36**
**SA**	**lateraloccipital**	**rs3808477**	**T**	**C**	**T**	**C**	**0.0174**	**0.0025**	**3.30E-12**	**20**	**-4.7581**	**3.7521**	**0.2048**	**0.28**
**SA**	**lateraloccipital**	**rs4241964**	**G**	**T**	**G**	**T**	**0.0160**	**0.0023**	**1.41E-12**	**25**	**4.2403**	**3.5084**	**0.2268**	**0.48**
**SA**	**lateraloccipital**	**rs4245555**	**C**	**T**	**C**	**T**	**0.0173**	**0.0023**	**6.13E-14**	**27**	**1.9631**	**3.4537**	**0.5698**	**0.41**
**SA**	**lateraloccipital**	**rs4549082**	**C**	**T**	**C**	**T**	**0.0170**	**0.0022**	**3.61E-14**	**29**	**9.3585**	**3.6853**	**0.0111**	**0.48**
**SA**	**lateraloccipital**	**rs4550384**	**G**	**T**	**G**	**T**	**-0.0146**	**0.0026**	**2.49E-08**	**11**	**-9.2436**	**3.9886**	**0.0205**	**0.24**
**SA**	**lateraloccipital**	**rs4588790**	**T**	**C**	**T**	**C**	**-0.0130**	**0.0023**	**1.84E-08**	**15**	**0.4917**	**3.4597**	**0.8870**	**0.38**
**SA**	**lateraloccipital**	**rs4622900**	**C**	**T**	**C**	**T**	**-0.0125**	**0.0023**	**2.99E-08**	**15**	**0.7807**	**3.4237**	**0.8196**	**0.44**
**SA**	**lateraloccipital**	**rs4899502**	**G**	**A**	**G**	**A**	**0.0136**	**0.0024**	**1.83E-08**	**14**	**0.6097**	**3.6580**	**0.8676**	**0.31**
**SA**	**lateraloccipital**	**rs4936289**	**G**	**A**	**G**	**A**	**0.0131**	**0.0023**	**1.33E-08**	**15**	**3.3537**	**3.5880**	**0.3499**	**0.38**
**SA**	**lateraloccipital**	**rs55910701**	**T**	**C**	**T**	**C**	**-0.0179**	**0.0026**	**1.05E-11**	**17**	**4.6873**	**4.0095**	**0.2424**	**0.24**
**SA**	**lateraloccipital**	**rs56049037**	**A**	**G**	**A**	**G**	**-0.0159**	**0.0025**	**1.64E-10**	**17**	**8.0244**	**4.0757**	**0.0490**	**0.29**
**SA**	**lateraloccipital**	**rs56076457**	**C**	**T**	**C**	**T**	**-0.0127**	**0.0022**	**1.58E-08**	**16**	**-3.6824**	**3.3998**	**0.2788**	**0.47**
**SA**	**lateraloccipital**	**rs6131942**	**A**	**G**	**A**	**G**	**-0.0136**	**0.0023**	**2.31E-09**	**17**	**0.3497**	**3.4502**	**0.9193**	**0.42**
**SA**	**lateraloccipital**	**rs61773390**	**T**	**G**	**T**	**G**	**0.0258**	**0.0028**	**5.34E-20**	**26**	**4.2734**	**4.3153**	**0.3220**	**0.20**
**SA**	**lateraloccipital**	**rs62172117**	**A**	**G**	**A**	**G**	**-0.0159**	**0.0023**	**1.08E-11**	**21**	**-1.5655**	**3.5550**	**0.6597**	**0.36**
**SA**	**lateraloccipital**	**rs62182115**	**T**	**C**	**T**	**C**	**-0.0135**	**0.0024**	**1.04E-08**	**15**	**2.0047**	**3.7098**	**0.5889**	**0.34**
**SA**	**lateraloccipital**	**rs6441371**	**A**	**C**	**A**	**C**	**0.0140**	**0.0023**	**5.54E-10**	**19**	**2.1101**	**3.3620**	**0.5303**	**0.48**
**SA**	**lateraloccipital**	**rs6778003**	**T**	**G**	**T**	**G**	**0.0152**	**0.0025**	**6.05E-10**	**16**	**0.6080**	**4.0412**	**0.8804**	**0.29**
**SA**	**lateraloccipital**	**rs6990077**	**T**	**C**	**T**	**C**	**-0.0185**	**0.0027**	**7.90E-12**	**16**	**4.5834**	**4.1440**	**0.2687**	**0.22**
**SA**	**lateraloccipital**	**rs7196720**	**T**	**C**	**T**	**C**	**0.0125**	**0.0022**	**2.23E-08**	**16**	**3.7591**	**3.4539**	**0.2764**	**0.49**
**SA**	**lateraloccipital**	**rs72720396**	**G**	**A**	**G**	**A**	**0.0223**	**0.0027**	**4.36E-17**	**25**	**3.0619**	**4.1952**	**0.4655**	**0.23**
**SA**	**lateraloccipital**	**rs7304278**	**A**	**G**	**A**	**G**	**-0.0149**	**0.0025**	**3.59E-09**	**14**	**-3.2783**	**3.7555**	**0.3827**	**0.28**
**SA**	**lateraloccipital**	**rs73095857**	**T**	**G**	**T**	**G**	**0.0134**	**0.0023**	**3.52E-09**	**17**	**5.2471**	**3.5778**	**0.1425**	**0.42**
**SA**	**lateraloccipital**	**rs7313852**	**A**	**G**	**A**	**G**	**-0.0226**	**0.0023**	**2.52E-23**	**49**	**-6.2528**	**3.5420**	**0.0775**	**0.44**

**SA**	**lateraloccipital**	**rs735459**	**C**	**T**	**C**	**T**	**0.0155**	**0.0024**	**5.25E-11**	**20**	**1.0928**	**3.6061**	**0.7619**	**0.36**
**SA**	**lateraloccipital**	**rs7598252**	**G**	**A**	**G**	**A**	**-0.0128**	**0.0023**	**2.00E-08**	**16**	**3.5042**	**3.4098**	**0.3041**	**0.47**
**SA**	**lateraloccipital**	**rs7783012**	**G**	**A**	**G**	**A**	**-0.0140**	**0.0023**	**1.09E-09**	**18**	**-2.0576**	**3.4517**	**0.5511**	**0.41**
**SA**	**lateraloccipital**	**rs77960**	**A**	**G**	**A**	**G**	**0.0136**	**0.0024**	**1.22E-08**	**14**	**-3.2358**	**3.6609**	**0.3768**	**0.33**
**SA**	**lateraloccipital**	**rs7925560**	**A**	**G**	**A**	**G**	**-0.0173**	**0.0027**	**6.54E-11**	**15**	**-6.8496**	**3.9633**	**0.0839**	**0.23**
**SA**	**lateraloccipital**	**rs7959983**	**C**	**T**	**C**	**T**	**0.0151**	**0.0023**	**3.17E-11**	**21**	**-5.1883**	**3.4612**	**0.1339**	**0.40**
**SA**	**lateraloccipital**	**rs8072507**	**C**	**T**	**C**	**T**	**0.0150**	**0.0025**	**1.37E-09**	**15**	**-2.5651**	**3.6839**	**0.4862**	**0.29**
**SA**	**lateraloccipital**	**rs9348050**	**T**	**C**	**T**	**C**	**0.0133**	**0.0022**	**3.44E-09**	**17**	**-6.4360**	**3.4459**	**0.0618**	**0.49**
**SA**	**lateraloccipital**	**rs9395520**	**T**	**C**	**T**	**C**	**0.0180**	**0.0024**	**1.59E-13**	**23**	**-2.1889**	**3.6552**	**0.5493**	**0.30**
**SA**	**lateraloccipital**	**rs9597250**	**A**	**C**	**A**	**C**	**-0.0178**	**0.0029**	**6.12E-10**	**12**	**4.1348**	**4.2677**	**0.3326**	**0.19**
**SA**	**lateraloccipital**	**rs974552**	**G**	**A**	**G**	**A**	**0.0192**	**0.0028**	**1.48E-11**	**14**	**4.8232**	**4.5442**	**0.2885**	**0.19**
**SA**	**lateraloccipital**	**rs9795439**	**A**	**G**	**A**	**G**	**0.0163**	**0.0028**	**6.68E-09**	**11**	**5.6156**	**4.9745**	**0.2590**	**0.20**
**SA**	**lateraloccipital**	**rs9964420**	**A**	**C**	**A**	**C**	**-0.0202**	**0.0025**	**2.46E-16**	**28**	**-2.3718**	**3.7703**	**0.5293**	**0.30**
**TH**	**frontalpole**	**rs10518440**	**G**	**A**	**G**	**A**	**0.0310**	**0.0030**	**2.07E-24**	**28**	**-0.0019**	**0.0027**	**0.4754**	**0.16**
**TH**	**frontalpole**	**rs10737452**	**C**	**T**	**C**	**T**	**0.0159**	**0.0023**	**6.30E-12**	**22**	**-0.0021**	**0.0021**	**0.3044**	**0.38**
**TH**	**frontalpole**	**rs112439757**	**T**	**C**	**T**	**C**	**-0.0219**	**0.0029**	**6.57E-14**	**17**	**-0.0017**	**0.0026**	**0.5200**	**0.18**
**TH**	**frontalpole**	**rs12206116**	**A**	**G**	**A**	**G**	**0.0126**	**0.0023**	**4.40E-08**	**14**	**-0.0030**	**0.0020**	**0.1426**	**0.40**
**TH**	**frontalpole**	**rs12431576**	**C**	**T**	**C**	**T**	**-0.0148**	**0.0024**	**7.63E-10**	**17**	**0.0009**	**0.0022**	**0.6690**	**0.33**
**TH**	**frontalpole**	**rs12927162**	**G**	**A**	**G**	**A**	**-0.0234**	**0.0025**	**9.89E-21**	**35**	**0.0039**	**0.0025**	**0.1186**	**0.28**
**TH**	**frontalpole**	**rs12956276**	**A**	**G**	**A**	**G**	**0.0132**	**0.0023**	**1.45E-08**	**15**	**0.0019**	**0.0021**	**0.3636**	**0.35**
**TH**	**frontalpole**	**rs1323591**	**T**	**C**	**T**	**C**	**-0.0143**	**0.0024**	**1.64E-09**	**16**	**0.0027**	**0.0022**	**0.2208**	**0.32**
**TH**	**frontalpole**	**rs139911**	**C**	**T**	**C**	**T**	**0.0183**	**0.0023**	**9.94E-16**	**31**	**-0.0028**	**0.0020**	**0.1633**	**0.42**
**TH**	**frontalpole**	**rs1421085**	**C**	**T**	**C**	**T**	**0.0215**	**0.0023**	**4.08E-21**	**43**	**-0.0043**	**0.0020**	**0.0321**	**0.40**
**TH**	**frontalpole**	**rs17448682**	**T**	**C**	**T**	**C**	**0.0160**	**0.0027**	**1.75E-09**	**13**	**0.0015**	**0.0024**	**0.5238**	**0.23**
**TH**	**frontalpole**	**rs17697212**	**C**	**T**	**C**	**T**	**-0.0162**	**0.0028**	**1.02E-08**	**10**	**0.0020**	**0.0025**	**0.4254**	**0.20**

**TH**	**frontalpole**	**rs202157**	**C**	**T**	**C**	**T**	**0.0193**	**0.0025**	**4.42E-15**	**26**	**0.0008**	**0.0022**	**0.7114**	**0.30**
**TH**	**frontalpole**	**rs2232839**	**T**	**C**	**T**	**C**	**-0.0211**	**0.0028**	**4.81E-14**	**19**	**-0.0003**	**0.0028**	**0.9044**	**0.21**
**TH**	**frontalpole**	**rs2291589**	**G**	**T**	**G**	**T**	**-0.0165**	**0.0023**	**8.13E-13**	**24**	**0.0018**	**0.0021**	**0.3887**	**0.38**
**TH**	**frontalpole**	**rs2653349**	**A**	**G**	**A**	**G**	**0.0295**	**0.0027**	**4.50E-27**	**39**	**-0.0004**	**0.0025**	**0.8701**	**0.21**
**TH**	**frontalpole**	**rs2701524**	**C**	**T**	**C**	**T**	**-0.0126**	**0.0023**	**4.36E-08**	**15**	**0.0025**	**0.0021**	**0.2304**	**0.41**
**TH**	**frontalpole**	**rs2794682**	**T**	**C**	**T**	**C**	**0.0170**	**0.0023**	**9.34E-14**	**27**	**0.0015**	**0.0021**	**0.4582**	**0.40**
**TH**	**frontalpole**	**rs2881955**	**T**	**C**	**T**	**C**	**0.0144**	**0.0025**	**8.59E-09**	**13**	**-0.0007**	**0.0022**	**0.7664**	**0.28**
**TH**	**frontalpole**	**rs2916142**	**T**	**C**	**T**	**C**	**0.0135**	**0.0023**	**4.41E-09**	**17**	**-0.0026**	**0.0020**	**0.1977**	**0.46**
**TH**	**frontalpole**	**rs3100052**	**A**	**G**	**A**	**G**	**0.0126**	**0.0023**	**4.35E-08**	**14**	**-0.0016**	**0.0021**	**0.4289**	**0.39**
**TH**	**frontalpole**	**rs34571177**	**G**	**T**	**G**	**T**	**-0.0174**	**0.0029**	**2.68E-09**	**10**	**-0.0002**	**0.0026**	**0.9354**	**0.18**
**TH**	**frontalpole**	**rs35376824**	**G**	**A**	**G**	**A**	**-0.0161**	**0.0026**	**1.12E-09**	**13**	**0.0036**	**0.0024**	**0.1348**	**0.23**
**TH**	**frontalpole**	**rs35935113**	**C**	**T**	**C**	**T**	**-0.0124**	**0.0023**	**3.59E-08**	**15**	**0.0012**	**0.0020**	**0.5407**	**0.45**
**TH**	**frontalpole**	**rs36117029**	**G**	**A**	**G**	**A**	**0.0129**	**0.0024**	**3.76E-08**	**14**	**0.0027**	**0.0021**	**0.2002**	**0.36**
**TH**	**frontalpole**	**rs3808477**	**T**	**C**	**T**	**C**	**0.0174**	**0.0025**	**3.30E-12**	**20**	**-0.0004**	**0.0022**	**0.8512**	**0.28**
**TH**	**frontalpole**	**rs4241964**	**G**	**T**	**G**	**T**	**0.0160**	**0.0023**	**1.41E-12**	**25**	**-0.0007**	**0.0021**	**0.7299**	**0.48**
**TH**	**frontalpole**	**rs4245555**	**C**	**T**	**C**	**T**	**0.0173**	**0.0023**	**6.13E-14**	**27**	**0.0048**	**0.0020**	**0.0170**	**0.41**
**TH**	**frontalpole**	**rs4549082**	**C**	**T**	**C**	**T**	**0.0170**	**0.0022**	**3.61E-14**	**29**	**-0.0013**	**0.0021**	**0.5549**	**0.48**
**TH**	**frontalpole**	**rs4550384**	**G**	**T**	**G**	**T**	**-0.0146**	**0.0026**	**2.49E-08**	**11**	**-0.0005**	**0.0023**	**0.8386**	**0.24**
**TH**	**frontalpole**	**rs4588790**	**T**	**C**	**T**	**C**	**-0.0130**	**0.0023**	**1.84E-08**	**15**	**-0.0020**	**0.0020**	**0.3234**	**0.38**
**TH**	**frontalpole**	**rs4622900**	**C**	**T**	**C**	**T**	**-0.0125**	**0.0023**	**2.99E-08**	**15**	**0.0013**	**0.0020**	**0.5317**	**0.44**
**TH**	**frontalpole**	**rs4899502**	**G**	**A**	**G**	**A**	**0.0136**	**0.0024**	**1.83E-08**	**14**	**0.0017**	**0.0021**	**0.4290**	**0.31**
**TH**	**frontalpole**	**rs4936289**	**G**	**A**	**G**	**A**	**0.0131**	**0.0023**	**1.33E-08**	**15**	**-0.0024**	**0.0021**	**0.2484**	**0.38**
**TH**	**frontalpole**	**rs55910701**	**T**	**C**	**T**	**C**	**-0.0179**	**0.0026**	**1.05E-11**	**17**	**-0.0013**	**0.0023**	**0.5734**	**0.24**
**TH**	**frontalpole**	**rs56049037**	**A**	**G**	**A**	**G**	**-0.0159**	**0.0025**	**1.64E-10**	**17**	**0.0018**	**0.0024**	**0.4534**	**0.29**
**TH**	**frontalpole**	**rs56076457**	**C**	**T**	**C**	**T**	**-0.0127**	**0.0022**	**1.58E-08**	**16**	**0.0006**	**0.0020**	**0.7768**	**0.47**

**TH**	**frontalpole**	**rs6131942**	**A**	**G**	**A**	**G**	**-0.0136**	**0.0023**	**2.31E-09**	**17**	**-0.0012**	**0.0020**	**0.5436**	**0.42**
**TH**	**frontalpole**	**rs61773390**	**T**	**G**	**T**	**G**	**0.0258**	**0.0028**	**5.34E-20**	**26**	**-0.0017**	**0.0025**	**0.5035**	**0.20**
**TH**	**frontalpole**	**rs62172117**	**A**	**G**	**A**	**G**	**-0.0159**	**0.0023**	**1.08E-11**	**21**	**-0.0003**	**0.0021**	**0.8694**	**0.36**
**TH**	**frontalpole**	**rs62182115**	**T**	**C**	**T**	**C**	**-0.0135**	**0.0024**	**1.04E-08**	**15**	**-0.0011**	**0.0022**	**0.5969**	**0.34**
**TH**	**frontalpole**	**rs6441371**	**A**	**C**	**A**	**C**	**0.0140**	**0.0023**	**5.54E-10**	**19**	**-0.0004**	**0.0020**	**0.8539**	**0.48**
**TH**	**frontalpole**	**rs6778003**	**T**	**G**	**T**	**G**	**0.0152**	**0.0025**	**6.05E-10**	**16**	**0.0029**	**0.0023**	**0.2201**	**0.29**
**TH**	**frontalpole**	**rs6990077**	**T**	**C**	**T**	**C**	**-0.0185**	**0.0027**	**7.90E-12**	**16**	**0.0000**	**0.0024**	**0.9889**	**0.22**
**TH**	**frontalpole**	**rs7196720**	**T**	**C**	**T**	**C**	**0.0125**	**0.0022**	**2.23E-08**	**16**	**0.0001**	**0.0020**	**0.9688**	**0.49**
**TH**	**frontalpole**	**rs7304278**	**A**	**G**	**A**	**G**	**-0.0149**	**0.0025**	**3.59E-09**	**14**	**-0.0008**	**0.0022**	**0.7120**	**0.28**
**TH**	**frontalpole**	**rs73095857**	**T**	**G**	**T**	**G**	**0.0134**	**0.0023**	**3.52E-09**	**17**	**-0.0004**	**0.0021**	**0.8622**	**0.42**
**TH**	**frontalpole**	**rs7313852**	**A**	**G**	**A**	**G**	**-0.0226**	**0.0023**	**2.52E-23**	**49**	**0.0024**	**0.0021**	**0.2579**	**0.44**
**TH**	**frontalpole**	**rs735459**	**C**	**T**	**C**	**T**	**0.0155**	**0.0024**	**5.25E-11**	**20**	**-0.0004**	**0.0021**	**0.8682**	**0.36**
**TH**	**frontalpole**	**rs7598252**	**G**	**A**	**G**	**A**	**-0.0128**	**0.0023**	**2.00E-08**	**16**	**0.0011**	**0.0020**	**0.5655**	**0.47**
**TH**	**frontalpole**	**rs7783012**	**G**	**A**	**G**	**A**	**-0.0140**	**0.0023**	**1.09E-09**	**18**	**0.0013**	**0.0020**	**0.5240**	**0.41**
**TH**	**frontalpole**	**rs77960**	**A**	**G**	**A**	**G**	**0.0136**	**0.0024**	**1.22E-08**	**14**	**-0.0006**	**0.0021**	**0.7705**	**0.33**
**TH**	**frontalpole**	**rs8072507**	**C**	**T**	**C**	**T**	**0.0150**	**0.0025**	**1.37E-09**	**15**	**0.0027**	**0.0022**	**0.2031**	**0.29**
**TH**	**frontalpole**	**rs9348050**	**T**	**C**	**T**	**C**	**0.0133**	**0.0022**	**3.44E-09**	**17**	**-0.0053**	**0.0020**	**0.0091**	**0.49**
**TH**	**frontalpole**	**rs9395520**	**T**	**C**	**T**	**C**	**0.0180**	**0.0024**	**1.59E-13**	**23**	**0.0014**	**0.0021**	**0.5005**	**0.30**
**TH**	**frontalpole**	**rs9597250**	**A**	**C**	**A**	**C**	**-0.0178**	**0.0029**	**6.12E-10**	**12**	**-0.0012**	**0.0025**	**0.6253**	**0.19**
**TH**	**frontalpole**	**rs974552**	**G**	**A**	**G**	**A**	**0.0192**	**0.0028**	**1.48E-11**	**14**	**-0.0035**	**0.0027**	**0.1904**	**0.19**
**TH**	**frontalpole**	**rs9795439**	**A**	**G**	**A**	**G**	**0.0163**	**0.0028**	**6.68E-09**	**11**	**-0.0006**	**0.0029**	**0.8482**	**0.20**
**TH**	**frontalpole**	**rs9964420**	**A**	**C**	**A**	**C**	**-0.0202**	**0.0025**	**2.46E-16**	**28**	**0.0033**	**0.0022**	**0.1377**	**0.30**

Table S2. The details of genetic instruments predicting morningness and cortical SA/TH

The details of genetic instruments predicting insomnia and cortical SA/TH														
cortical SA/TH	Exposure	SNP	EA.exposure	OA.exposure	EA.outcome	other_allele.outcome	beta.exposure	se.exposure	pval.exposure	F	beta.outcome	se.outcome	pval.outcome	MAF
TH	parahippocampal	rs11693221	T	C	T	C	1.1310	0.0127	3.14E-22	634	0.0090	0.0072	0.2093	0.04
TH	parahippocampal	rs1456193	T	C	T	C	0.9633	0.0067	2.13E-08	6248	0.0011	0.0031	0.7290	0.18
TH	parahippocampal	rs370771	G	T	G	T	0.9660	0.0051	1.48E-11	18484	-0.0007	0.0025	0.7856	0.45
TH	parahippocampal	rs4073582	A	G	A	G	0.9693	0.0053	4.67E-09	15911	-0.0004	0.0025	0.8810	0.36
TH	parahippocampal	rs4986172	T	C	T	C	1.0380	0.0054	5.20E-12	17670	0.0034	0.0025	0.1689	0.34
TH	parahippocampal	rs55683518	G	T	G	T	0.9711	0.0053	3.55E-08	16171	0.0047	0.0024	0.0560	0.37
TH	parahippocampal	rs6938026	G	A	G	A	1.0380	0.0062	2.72E-09	9384	0.0002	0.0029	0.9466	0.21
TH	parahippocampal	rs6984111	C	T	C	T	1.0440	0.0074	4.25E-09	5455	0.0053	0.0033	0.1066	0.16
TH	parahippocampal	rs77217059	A	G	A	G	0.9592	0.0072	8.76E-09	4516	0.0008	0.0033	0.8179	0.15
TH	parahippocampal	rs77960	A	G	A	G	1.0330	0.0054	1.66E-09	16623	0.0032	0.0025	0.2090	0.33
TH	parahippocampal	rs9576155	A	G	A	G	1.0310	0.0054	9.26E-09	17169	-0.0014	0.0028	0.6174	0.34

Table S3. The details of genetic instruments predicting insomnia and cortical SA/TH

The details of genetic instruments predicting ease of getting up and cortical SA/TH														
cortical SA/TH	Exposure	SNP	EA.exposure	OA.exposure	EA.outcome	OA.outcome	beta.exposure	se.exposure	pval.exposure	F	beta.outcome	se.outcome	pval.outcome	MAF
SA	lateralorbitofrontal	rs10175975	T	C	T	C	0.0134	0.0023	3.30E-09	35	-3.5129	2.1682	0.1052	0.18
SA	lateralorbitofrontal	rs10470887	G	A	G	A	-0.0095	0.0017	4.05E-08	30	-0.2230	1.7015	0.8957	0.49
SA	lateralorbitofrontal	rs11229264	A	G	A	G	-0.0122	0.0019	1.43E-10	41	-3.7515	1.8451	0.0420	0.30
SA	lateralorbitofrontal	rs113240734	A	G	A	G	0.0192	0.0023	2.17E-16	67	4.1233	2.3012	0.0732	0.16
SA	lateralorbitofrontal	rs11642015	T	C	T	C	0.0115	0.0018	6.20E-11	43	0.9359	1.7257	0.5876	0.40
SA	lateralorbitofrontal	rs11643192	A	C	A	C	-0.0107	0.0018	1.30E-09	37	-2.7010	1.7405	0.1207	0.39
SA	lateralorbitofrontal	rs11697690	C	T	C	T	0.0097	0.0017	2.33E-08	31	2.1816	1.7192	0.2044	0.47
SA	lateralorbitofrontal	rs12515274	A	G	A	G	-0.0123	0.0020	5.37E-10	39	-1.8198	1.9625	0.3538	0.25
SA	lateralorbitofrontal	rs12601968	T	G	T	G	-0.0112	0.0018	1.16E-09	37	-0.0467	1.8366	0.9797	0.33
SA	lateralorbitofrontal	rs1420607	A	G	A	G	0.0131	0.0019	1.57E-11	45	1.7503	1.9530	0.3701	0.27

**SA**	**lateralorbitofrontal**	**rs1459192**	**T**	**C**	**T**	**C**	**-0.0102**	**0.0018**	**2.50E-08**	**31**	**-1.5453**	**1.7893**	**0.3878**	**0.33**
**SA**	**lateralorbitofrontal**	**rs1606803**	**T**	**C**	**T**	**C**	**0.0119**	**0.0019**	**3.22E-10**	**40**	**-0.9653**	**1.8843**	**0.6084**	**0.29**
**SA**	**lateralorbitofrontal**	**rs16917522**	**C**	**T**	**C**	**T**	**0.0136**	**0.0022**	**1.12E-09**	**37**	**1.7027**	**2.2180**	**0.4427**	**0.18**
**SA**	**lateralorbitofrontal**	**rs17822102**	**G**	**A**	**G**	**A**	**0.0111**	**0.0018**	**9.40E-10**	**37**	**1.2039**	**1.7972**	**0.5029**	**0.34**
**SA**	**lateralorbitofrontal**	**rs2037387**	**T**	**C**	**T**	**C**	**0.0104**	**0.0018**	**3.04E-09**	**35**	**-3.9475**	**1.7096**	**0.0209**	**0.42**
**SA**	**lateralorbitofrontal**	**rs2053457**	**C**	**T**	**C**	**T**	**-0.0160**	**0.0020**	**1.29E-15**	**64**	**-0.6499**	**1.9463**	**0.7385**	**0.25**
**SA**	**lateralorbitofrontal**	**rs2193749**	**T**	**C**	**T**	**C**	**-0.0097**	**0.0017**	**1.84E-08**	**32**	**2.2765**	**1.7187**	**0.1853**	**0.49**
**SA**	**lateralorbitofrontal**	**rs2653349**	**A**	**G**	**A**	**G**	**0.0234**	**0.0021**	**8.45E-29**	**124**	**3.8827**	**2.1086**	**0.0656**	**0.21**
**SA**	**lateralorbitofrontal**	**rs3735478**	**T**	**G**	**T**	**G**	**-0.0107**	**0.0019**	**1.57E-08**	**32**	**-0.4544**	**1.9184**	**0.8128**	**0.29**
**SA**	**lateralorbitofrontal**	**rs3760185**	**T**	**C**	**T**	**C**	**-0.0111**	**0.0020**	**4.52E-08**	**30**	**-1.0921**	**2.1878**	**0.6177**	**0.24**
**SA**	**lateralorbitofrontal**	**rs406952**	**C**	**T**	**C**	**T**	**0.0101**	**0.0018**	**1.20E-08**	**33**	**2.8727**	**1.7403**	**0.0988**	**0.38**
**SA**	**lateralorbitofrontal**	**rs4483990**	**C**	**A**	**C**	**A**	**-0.0172**	**0.0024**	**6.40E-13**	**52**	**0.3771**	**2.3998**	**0.8751**	**0.15**
**SA**	**lateralorbitofrontal**	**rs4671328**	**T**	**G**	**T**	**G**	**0.0107**	**0.0018**	**1.06E-09**	**37**	**2.8054**	**1.7354**	**0.1060**	**0.45**

**SA**	**lateralorbitofrontal**	**rs4853283**	**G**	**A**	**G**	**A**	**-0.0143**	**0.0017**	**1.84E-16**	**68**	**-2.7920**	**1.7095**	**0.1024**	**0.44**
**SA**	**lateralorbitofrontal**	**rs4884166**	**A**	**G**	**A**	**G**	**-0.0123**	**0.0022**	**1.15E-08**	**33**	**-2.7210**	**2.1213**	**0.1996**	**0.20**
**SA**	**lateralorbitofrontal**	**rs4958316**	**A**	**C**	**A**	**C**	**0.0150**	**0.0019**	**3.69E-15**	**62**	**0.3036**	**1.8740**	**0.8713**	**0.29**
**SA**	**lateralorbitofrontal**	**rs61773374**	**G**	**A**	**G**	**A**	**0.0165**	**0.0022**	**3.37E-14**	**58**	**-3.3091**	**2.1724**	**0.1277**	**0.20**
**SA**	**lateralorbitofrontal**	**rs6575012**	**A**	**G**	**A**	**G**	**-0.0096**	**0.0017**	**2.57E-08**	**31**	**1.1691**	**1.7044**	**0.4927**	**0.48**
**SA**	**lateralorbitofrontal**	**rs6581138**	**A**	**G**	**A**	**G**	**0.0127**	**0.0020**	**2.30E-10**	**40**	**-2.3690**	**2.0752**	**0.2536**	**0.24**
**SA**	**lateralorbitofrontal**	**rs7297799**	**T**	**C**	**T**	**C**	**-0.0128**	**0.0018**	**1.21E-12**	**50**	**0.2444**	**1.8364**	**0.8941**	**0.40**
**SA**	**lateralorbitofrontal**	**rs76048411**	**T**	**C**	**T**	**C**	**0.0106**	**0.0017**	**8.53E-10**	**38**	**-0.0079**	**1.8096**	**0.9965**	**0.48**
**SA**	**lateralorbitofrontal**	**rs77556405**	**A**	**G**	**A**	**G**	**0.0168**	**0.0023**	**1.59E-13**	**54**	**-1.2042**	**2.4250**	**0.6195**	**0.17**
**SA**	**lateralorbitofrontal**	**rs77576965**	**T**	**C**	**T**	**C**	**0.0109**	**0.0019**	**1.94E-08**	**32**	**0.7692**	**1.8851**	**0.6832**	**0.27**
**SA**	**lateralorbitofrontal**	**rs9399613**	**T**	**C**	**T**	**C**	**-0.0109**	**0.0019**	**1.32E-08**	**32**	**-2.4681**	**1.9218**	**0.1991**	**0.29**
**SA**	**lateralorbitofrontal**	**rs9995419**	**A**	**G**	**A**	**G**	**0.0114**	**0.0020**	**1.50E-08**	**32**	**-1.0339**	**1.9769**	**0.6010**	**0.24**

Table S4. The details of genetic instruments predicting ease of getting up and cortical SA/TH

The details of genetic instruments predicting long sleep and cortical SA/TH														
**cortical SA/TH**	**Exposure**	**SNP**	**EA.exposure**	**OA.exposure**	**EA.outcome**	**OA.outcome**	**beta.exposure**	**se.exposure**	**pval.exposure**	**F**	**beta.outcome**	**se.outcome**	**pval.outcome**	**EAF**
**SA**	**isthmuscingulate**	**rs10899257**	**G**	**A**	**G**	**A**	**-0.0056**	**0.0010**	**4.60E-08**	**30**	**-2.0704**	**1.4808**	**0.1620**	**0.86**
**SA**	**isthmuscingulate**	**rs17817288**	**A**	**G**	**A**	**G**	**0.0042**	**0.0007**	**8.90E-09**	**33**	**0.7948**	**1.0195**	**0.4356**	**0.52**
**SA**	**isthmuscingulate**	**rs3751046**	**A**	**G**	**A**	**G**	**-0.0058**	**0.0010**	**2.00E-08**	**32**	**-0.2995**	**1.4518**	**0.8366**	**0.85**
**SA**	**isthmuscingulate**	**rs6737318**	**A**	**G**	**A**	**G**	**-0.0064**	**0.0009**	**3.40E-13**	**53**	**-3.2236**	**1.2655**	**0.0109**	**0.78**
**SA**	**isthmuscingulate**	**rs75458655**	**C**	**T**	**C**	**T**	**-0.0167**	**0.0024**	**5.40E-12**	**48**	**-1.7229**	**4.6509**	**0.7111**	**0.98**
**SA**	**parsopercularis**	**rs10899257**	**G**	**A**	**G**	**A**	**-0.0056**	**0.0010**	**4.60E-08**	**30**	**1.0671**	**2.3435**	**0.6489**	**0.86**
**SA**	**parsopercularis**	**rs17817288**	**A**	**G**	**A**	**G**	**0.0042**	**0.0007**	**8.90E-09**	**33**	**2.4141**	**1.6134**	**0.1346**	**0.52**
**SA**	**parsopercularis**	**rs3751046**	**A**	**G**	**A**	**G**	**-0.0058**	**0.0010**	**2.00E-08**	**32**	**-3.2150**	**2.2963**	**0.1615**	**0.85**
**SA**	**parsopercularis**	**rs6737318**	**A**	**G**	**A**	**G**	**-0.0064**	**0.0009**	**3.40E-13**	**53**	**-3.8391**	**2.0029**	**0.0553**	**0.78**
**SA**	**parsopercularis**	**rs75458655**	**C**	**T**	**C**	**T**	**-0.0167**	**0.0024**	**5.40E-12**	**48**	**-5.0283**	**7.4024**	**0.4970**	**0.98**

Table S5. The details of genetic instruments predicting long sleep and cortical SA/TH

The Association of sleep traits and cortical SA/TH									
**Outcome**	**Cortical SA/TH**	**Exposure**	**Method**	**NSNP**	**Beta**	**SE**	**Pval**	**Lowci95**	**Upci95**
**Morning**	**SA**	**lateraloccipital**	**MR Egger**	**67**	**109.1841**	**146.4568**	**0.4587**	**-374.5959**	**277.5089**
**Morning**	**SA**	**lateraloccipital**	**Weighted median**	**67**	**118.8897**	**42.6079**	**0.0053**	**-183.6778**	**11.2631**
**Morning**	**SA**	**lateraloccipital**	**Inverse variance weighted**	**67**	**88.9982**	**31.8073**	**0.0051**	**-150.4952**	**-4.8863**
**Morning**	**SA**	**lateraloccipital**	**Simple mode**	**67**	**183.8995**	**105.7309**	**0.0866**	**-309.4185**	**134.2587**

**Morning**	**SA**	**lateraloccipital**	**Weighted mode**	**67**	**163.7421**	**85.9640**	**0.0612**	**-280.1732**	**129.1900**
**Morning**	**SA**	**inferiorparietal**	**MR Egger**	**59**	**-48.5435**	**166.3533**	**0.7715**	**-374.5959**	**277.5089**
**Morning**	**SA**	**inferiorparietal**	**Weighted median**	**59**	**-86.2074**	**49.8433**	**0.0837**	**-183.9002**	**11.4855**
**Morning**	**SA**	**inferiorparietal**	**Inverse variance weighted**	**59**	**-77.6908**	**37.1451**	**0.0365**	**-150.4952**	**-4.8863**
**Morning**	**SA**	**inferiorparietal**	**Simple mode**	**59**	**-87.5799**	**117.3736**	**0.4586**	**-317.6321**	**142.4723**
**Morning**	**SA**	**inferiorparietal**	**Weighted mode**	**59**	**-75.4916**	**106.3755**	**0.4808**	**-283.9876**	**133.0044**
**Morning**	**SA**	**cuneus**	**MR Egger**	**67**	**21.5314**	**52.3210**	**0.6820**	**-81.0177**	**124.0805**
**Morning**	**SA**	**cuneus**	**Weighted median**	**67**	**31.9273**	**15.9365**	**0.0451**	**0.6917**	**63.1628**
**Morning**	**SA**	**cuneus**	**Inverse variance weighted**	**67**	**32.6252**	**11.3640**	**0.0041**	**10.3518**	**54.8986**
**Morning**	**SA**	**cuneus**	**Simple mode**	**67**	**18.7639**	**38.2628**	**0.6255**	**-56.2312**	**93.7591**
**Morning**	**SA**	**cuneus**	**Weighted mode**	**67**	**18.7639**	**33.5967**	**0.5784**	**-47.0857**	**84.6135**
**Morning**	**TH**	**frontalpole**	**MR Egger**	**59**	**-0.0678**	**0.0769**	**0.3818**	**-0.2186**	**0.0830**
**Morning**	**TH**	**frontalpole**	**Weighted median**	**59**	**-0.0445**	**0.0255**	**0.0808**	**-0.0945**	**0.0055**
**Morning**	**TH**	**frontalpole**	**Inverse variance weighted**	**59**	**-0.0388**	**0.0173**	**0.0248**	**-0.0727**	**-0.0049**
**Morning**	**TH**	**frontalpole**	**Simple mode**	**59**	**-0.0526**	**0.0565**	**0.3559**	**-0.1634**	**0.0582**
**Morning**	**TH**	**frontalpole**	**Weighted mode**	**59**	**-0.0545**	**0.0548**	**0.3240**	**-0.1619**	**0.0529**
**Shortsleep**	**TH**	**frontalpole**	**MR Egger**	**20**	**-0.6244**	**0.3622**	**0.1019**	**-1.3344**	**0.0856**
**Shortsleep**	**TH**	**frontalpole**	**Weighted median**	**20**	**-0.1337**	**0.1062**	**0.2081**	**-0.3418**	**0.0745**
**Shortsleep**	**TH**	**frontalpole**	**Inverse variance weighted**	**20**	**-0.1793**	**0.0764**	**0.0189**	**-0.3290**	**-0.0296**
**Shortsleep**	**TH**	**frontalpole**	**Simple mode**	**20**	**-0.1293**	**0.2099**	**0.5451**	**-0.5407**	**0.2821**
**Shortsleep**	**TH**	**frontalpole**	**Weighted mode**	**20**	**-0.1360**	**0.2080**	**0.5211**	**-0.5437**	**0.2717**
**Shortsleep**	**SA**	**inferiorparietal_**	**MR Egger**	**20**	**-1138.9697**	**702.8486**	**0.1225**	**-2516.5529**	**238.6135**
**Shortsleep**	**SA**	**inferiorparietal_**	**Weighted median**	**20**	**-291.6344**	**205.1888**	**0.1552**	**-693.8046**	**110.5357**
**Shortsleep**	**SA**	**inferiorparietal_**	**Inverse variance weighted**	**20**	**-329.5500**	**147.4514**	**0.0254**	**-618.5548**	**-40.5453**
**Shortsleep**	**SA**	**inferiorparietal_**	**Simple mode**	**20**	**-224.8782**	**389.7923**	**0.5708**	**-988.8711**	**539.1147**

Shortsleep	SA	inferiorparietal_	Weighted mode	20	-254.7046	380.6179	0.5114	-1000.7157	491.3064
Shortsleep	SA	lateraloccipital	MR Egger	17	672.9334	741.1285	0.3782	-779.6785	2125.5452
Shortsleep	SA	lateraloccipital	Weighted median	17	423.8984	196.4640	0.0310	38.8289	808.9678
Shortsleep	SA	lateraloccipital	Inverse variance weighted	17	394.3693	146.1629	0.0070	107.8900	680.8486
Shortsleep	SA	lateraloccipital	Simple mode	17	663.9841	388.0322	0.1064	-96.5589	1424.5271
Shortsleep	SA	lateraloccipital	Weighted mode	17	465.7023	335.3087	0.1839	-191.5027	1122.9073
Shortsleep	SA	middletemporal_	MR Egger	16	661.5468	515.5059	0.2202	-348.8446	1671.9383
Shortsleep	SA	middletemporal_	Weighted median	16	292.7147	128.5795	0.0228	40.6988	544.7305
Shortsleep	SA	middletemporal_	Inverse variance weighted	16	200.1703	95.5122	0.0361	12.9664	387.3742
Shortsleep	SA	middletemporal_	Simple mode	16	316.5569	209.4736	0.1515	-94.0114	727.1252
Shortsleep	SA	middletemporal_	Weighted mode	16	307.3440	194.3347	0.1346	-73.5521	688.2401
Shortsleep	TH	middletemporal_	MR Egger	17	0.2433	0.1780	0.1918	-0.1056	0.5922
Shortsleep	TH	middletemporal_	Weighted median	17	0.1375	0.0538	0.0106	0.0320	0.2430
Shortsleep	TH	middletemporal_	Inverse variance weighted	17	0.1210	0.0388	0.0018	0.0450	0.1970
Shortsleep	TH	middletemporal_	Simple mode	17	0.1688	0.1019	0.1172	-0.0310	0.3686
Shortsleep	TH	middletemporal_	Weighted mode	17	0.1524	0.0978	0.1386	-0.0392	0.3441
Shortsleep	TH	paracentral	MR Egger	17	0.2433	0.1780	0.1918	-0.1056	0.5922
Shortsleep	TH	paracentral	Weighted median	17	0.1375	0.0538	0.0106	0.0320	0.2430
Shortsleep	TH	paracentral	Inverse variance weighted	17	0.1210	0.0388	0.0018	0.0450	0.1970
Shortsleep	TH	paracentral	Simple mode	17	0.1688	0.1019	0.1172	-0.0310	0.3686
Shortsleep	TH	paracentral	Weighted mode	17	0.1524	0.0978	0.1386	-0.0392	0.3441
Shortsleep	TH	parahippocampal	MR Egger	20	-0.1440	0.4301	0.7416	-0.9869	0.6989
Shortsleep	TH	parahippocampal	Weighted median	20	-0.2090	0.1194	0.0801	-0.4430	0.0251
Shortsleep	TH	parahippocampal	Inverse variance weighted	20	-0.2472	0.0904	0.0063	-0.4244	-0.0699
Shortsleep	TH	parahippocampal	Simple mode	20	-0.1933	0.2128	0.3751	-0.6105	0.2238

**Shortsleep**	**TH**	**parahippocampal**	**Weighted mode**	**20**	**-0.1428**	**0.1968**	**0.4769**	**-0.5286**	**0.2430**
**Shortsleep**	**TH**	**superiortemporal**	**MR Egger**	**20**	**0.2165**	**0.1741**	**0.2298**	**-0.1248**	**0.5578**
**Shortsleep**	**TH**	**superiortemporal**	**Weighted median**	**20**	**0.1506**	**0.0518**	**0.0036**	**0.0491**	**0.2521**
**Shortsleep**	**TH**	**superiortemporal**	**Inverse variance weighted**	**20**	**0.0900**	**0.0362**	**0.0130**	**0.0190**	**0.1611**
**Shortsleep**	**TH**	**superiortemporal**	**Simple mode**	**20**	**0.1722**	**0.0988**	**0.0974**	**-0.0214**	**0.3658**
**Shortsleep**	**TH**	**superiortemporal**	**Weighted mode**	**20**	**0.1705**	**0.1043**	**0.1186**	**-0.0339**	**0.3748**
**Insomnia**	**TH**	**parahippocampal**	**MR Egger**	**11**	**0.0233**	**0.0225**	**0.3272**	**-0.0208**	**0.0674**
**Insomnia**	**TH**	**parahippocampal**	**Weighted median**	**11**	**0.0011**	**0.0011**	**0.3266**	**-0.0011**	**0.0033**
**Insomnia**	**TH**	**parahippocampal**	**Inverse variance weighted**	**11**	**0.0018**	**0.0009**	**0.0374**	**0.0001**	**0.0034**
**Insomnia**	**TH**	**parahippocampal**	**Simple mode**	**11**	**0.0003**	**0.0020**	**0.8979**	**-0.0037**	**0.0043**
**Insomnia**	**TH**	**parahippocampal**	**Weighted mode**	**11**	**0.0002**	**0.0019**	**0.9204**	**-0.0035**	**0.0039**
**Ease of getting up**	**SA**	**lateralorbitofrontal**	**MR Egger**	**35**	**119.2731**	**126.3023**	**0.3519**	**-128.2794**	**366.8257**
**Ease of getting up**	**SA**	**lateralorbitofrontal**	**Weighted median**	**35**	**76.6210**	**37.9060**	**0.0432**	**2.3253**	**150.9167**
**Ease of getting up**	**SA**	**lateralorbitofrontal**	**Inverse variance weighted**	**35**	**57.6526**	**27.7510**	**0.0378**	**3.2606**	**112.0446**
**Ease of getting up**	**SA**	**lateralorbitofrontal**	**Simple mode**	**35**	**103.5609**	**81.9329**	**0.2148**	**-57.0277**	**264.1494**
**Ease of getting up**	**SA**	**lateralorbitofrontal**	**Weighted mode**	**35**	**139.2143**	**72.5811**	**0.0635**	**-3.0447**	**281.4734**
**Long sleep**	**SA**	**isthmuscingulate**	**MR Egger**	**5**	**228.6007**	**353.2287**	**0.5636**	**-463.7276**	**920.9290**
**Long sleep**	**SA**	**isthmuscingulate**	**Weighted median**	**5**	**234.1324**	**138.3513**	**0.0906**	**-37.0362**	**505.3010**
**Long sleep**	**SA**	**isthmuscingulate**	**Inverse variance weighted**	**5**	**274.9328**	**108.1136**	**0.0110**	**63.0301**	**486.8354**
**Long sleep**	**SA**	**isthmuscingulate**	**Simple mode**	**5**	**132.5394**	**196.5693**	**0.5371**	**-252.7364**	**517.8151**
**Long sleep**	**SA**	**isthmuscingulate**	**Weighted mode**	**5**	**134.8758**	**203.9808**	**0.5446**	**-264.9267**	**534.6782**
**Long sleep**	**SA**	**parsopercularis**	**MR Egger**	**5**	**252.8421**	**561.3255**	**0.6830**	**-847.3558**	**1353.0401**
**Long sleep**	**SA**	**parsopercularis**	**Weighted median**	**5**	**564.7283**	**205.0843**	**0.0059**	**162.7631**	**966.6934**
**Long sleep**	**SA**	**parsopercularis**	**Inverse variance weighted**	**5**	**409.0325**	**171.2302**	**0.0169**	**73.4213**	**744.6437**
**Long sleep**	**SA**	**parsopercularis**	**Simple mode**	**5**	**577.9232**	**306.1830**	**0.1321**	**-22.1955**	**1178.0419**

Long sleep	SA	parsopercularis	Weighted mode	5	582.0416	282.1081	0.1081	29.1098	1134.9734

Table S6. The Association of sleep traits and cortical SA/TH

The heterogeneity (cochrane's Q) of the mr results of sleep traits and cortical SA/TH					
cortical SA/TH	Outcome	Exposure	Method	Q	Q_pval
TH	Short sleep	frontalpole	MR Egger	15.1445	0.6520
TH	Short sleep	frontalpole	Inverse variance weighted	16.7243	0.6085
SA	Short sleep	inferiorparietal	MR Egger	15.6547	0.6166
SA	Short sleep	inferiorparietal	Inverse variance weighted	17.0420	0.5870
TH	Short sleep	parahippocampal	MR Egger	13.4554	0.7638
TH	Short sleep	parahippocampal	Inverse variance weighted	13.5156	0.8112
TH	Short sleep	paracentral	MR Egger	12.6652	0.6281
TH	Short sleep	paracentral	Inverse variance weighted	12.7070	0.6941
TH	Short sleep	middletemporal	MR Egger	18.0819	0.2584
TH	Short sleep	middletemporal	Inverse variance weighted	18.1950	0.3126
SA	Short sleep	middletemporal	MR Egger	10.7912	0.7024
SA	Short sleep	middletemporal	Inverse variance weighted	11.6207	0.7075
SA	Short sleep	lateraloccipital	MR Egger	17.5468	0.2872
SA	Short sleep	lateraloccipital	Inverse variance weighted	17.6965	0.3420
SA	morningness	cuneus	MR Egger	69.3552	0.1263
SA	morningness	cuneus	Inverse variance weighted	69.5536	0.1424
TH	morningness	frontalpole	MR Egger	49.9091	0.7359
TH	morningness	frontalpole	Inverse variance weighted	50.0587	0.7615
SA	morningness	lateraloccipital	MR Egger	73.2774	0.0720
SA	morningness	lateraloccipital	Inverse variance weighted	73.4735	0.0828

TH	insomnia	parahippocampal	MR Egger	6.7585	0.6622
TH	insomnia	parahippocampal	Inverse variance weighted	7.6764	0.6604
SA	Long sleep	isthmuscingulate	MR Egger	2.7453	0.4326
SA	Long sleep	isthmuscingulate	Inverse variance weighted	2.7643	0.5980
SA	Long sleep	parsopercularis	MR Egger	2.7502	0.4318
SA	Long sleep	parsopercularis	Inverse variance weighted	2.8355	0.5857
SA	Ease of getting up	lateralorbitofrontal	MR Egger	39.6765	0.1968
SA	Ease of getting up	lateralorbitofrontal	Inverse variance weighted	39.9775	0.2218

Table S7. The heterogeneity (cochrane's Q) of the mr results of sleep traits and cortical SA/TH

The Pleiotropy of the mr results of sleep traits and cortical SA/TH				
cortical SA/TH	Outcome	Exposure	egger_intercept	Pval
TH	Short sleep	frontalpole	0.003029	0.224855
TH	Short sleep	superiortemporal	-0.000859	0.467251
TH	Short sleep	parahippocampal	-0.000702	0.808923
TH	Short sleep	paracentral	-0.000296	0.840811
TH	Short sleep	middletemporal	-0.000397	0.763512
SA	Short sleep	middletemporal	-3.047812	0.377833
SA	Short sleep	inferiorparietal	5.500909	0.254200
SA	Short sleep	lateraloccipital	-1.778092	0.725543
SA	morningness	cuneus	0.374045	0.687865
TH	morningness	frontalpole	0.000490	0.700341
SA	morningness	lateraloccipital	0.956443	0.697633
TH	insomnia	parahippocampal	-0.021637	0.363065
SA	Long sleep	isthmuscingulate	0.281168	0.899142

**SA**	**Long sleep**	**lateralorbitofrontal**	**0.946796**	**0.789191**

Table S8. The Pleiotropy of the mr results of sleep traits and cortical SA/TH

Abbreviations: SA, Surficial area; TH, thickness.; IVs, Instrument variables; MR, Mendelian randomization; SNPs, Single-nucleotide polymorphisms.
